# MAPK pathway activity plays a key role in PD‐L1 expression of lung adenocarcinoma cells

**DOI:** 10.1002/path.5280

**Published:** 2019-05-21

**Authors:** Thijs S Stutvoet, Arjan Kol, Elisabeth GE de Vries, Marco de Bruyn, Rudolf SN Fehrmann, Anton GT Terwisscha van Scheltinga, Steven de Jong

**Affiliations:** ^1^ Department of Medical Oncology, Cancer Research Center Groningen University of Groningen, University Medical Center Groningen Groningen The Netherlands; ^2^ Department of Obstetrics and Gynecology, Cancer Research Center Groningen University of Groningen, University Medical Center Groningen Groningen The Netherlands; ^3^ Department of Clinical Pharmacy and Pharmacology University of Groningen, University Medical Center Groningen Groningen The Netherlands

**Keywords:** non‐small cell lung cancer (NSCLC), programmed death‐ligand 1 (PD‐L1), IFNγ, MAPK pathway, MHC‐I

## Abstract

Immune checkpoint inhibitors targeting programmed cell death protein 1 (PD‐1) and programmed death‐ligand 1 (PD‐L1) have improved the survival of patients with non‐small cell lung cancer (NSCLC). Still, many patients do not respond to these inhibitors. PD‐L1 (*CD274*) expression, one of the factors that influences the efficacy of immune checkpoint inhibitors, is dynamic. Here, we studied the regulation of PD‐L1 expression in NSCLC without targetable genetic alterations in *EGFR*, *ALK*, *BRAF*, *ROS1*, *MET*, *ERBB2* and *RET*. Analysis of RNA sequencing data from these NSCLCs revealed that inferred IFNγ, EGFR and MAPK signaling correlated with *CD274* gene expression in lung adenocarcinoma. In a representative lung adenocarcinoma cell line panel, stimulation with EGF or IFNγ increased *CD274* mRNA and PD‐L1 protein and membrane levels, which were further enhanced by combining EGF and IFNγ. Similarly, tumor cell PD‐L1 membrane levels increased after coculture with activated peripheral blood mononuclear cells. Inhibition of the MAPK pathway, using EGFR inhibitors cetuximab and erlotinib or the MEK 1 and 2 inhibitor selumetinib, prevented EGF‐ and IFNγ‐induced *CD274* mRNA and PD‐L1 protein and membrane upregulation, but had no effect on IFNγ‐induced MHC‐I upregulation. Interestingly, although IFNγ increases transcriptional activity of *CD274*, MAPK signaling also increased stabilization of *CD274* mRNA. In conclusion, MAPK pathway activity plays a key role in EGF‐ and IFNγ‐induced PD‐L1 expression in lung adenocarcinoma without targetable genetic alterations and may present a target to improve the efficacy of immunotherapy. © 2019 The Authors. Journal of Pathology published by John Wiley & Sons Ltd on behalf of Pathological Society of Great Britain and Ireland.

## Introduction

After years of limited progress in the treatment of advanced non‐small cell lung cancer (NSCLC), a major leap forward has been made with the introduction of programmed cell death protein 1 (PD‐1)/programmed death‐ligand 1 (PD‐L1) targeting immune checkpoint inhibitors. These have greatly improved the overall survival of patients with advanced NSCLC, especially patients without targetable genetic alterations, accounting for almost 60% of NSCLC 1, 2, 3. Patients with PD‐L1‐positive tumors generally respond better to PD‐1‐targeted immune checkpoint inhibition. However, discrepancies between observed PD‐L1 expression and the benefit from treatment often occur 4; even in a preselected patient population with >50% PD‐L1‐positive tumor cells, only 45–55% of patients respond to therapy 5. The limited value of tumor PD‐L1 as a biomarker may be caused by the highly dynamic expression of PD‐L1 due to the influence of multiple factors 6. The best characterized inducer of PD‐L1 expression in NSCLC is the pro‐inflammatory cytokine IFNγ, which is secreted by T cells 7, 8. PD‐L1 on tumor cells binds to PD‐1 on T cells, disrupting T cell function and thereby preventing an effective tumor immune response 9. Oncogenic driver mutations, such as mutations in *EGFR*, *ALK* and *BRAF*, are known inducers of PD‐L1 expression in NSCLC cells. In these oncogene‐activated cells, the PI3K/mTOR, JAK/STAT and MAPK pathways are the main drivers of PD‐L1 expression 10, 11, 12, 13.

Interestingly, *EGFR* wild‐type NSCLC tumors have higher levels of PD‐L1 and tumor infiltrating lymphocytes, and respond better to PD‐1/PD‐L1‐targeted therapy compared with *EGFR* mutant NSCLC 1, 2. However, there are only limited data about the regulation of PD‐L1 expression in NSCLC without targetable genetic alterations 14, 15, 16. A better understanding of PD‐L1 regulation may provide a rationale to combine immune checkpoint inhibitors with other targeted agents. In the present study, we aimed to identify pathways regulating *CD274* (PD‐L1) expression in this NSCLC subtype by using RNA sequencing data from The Cancer Genome Atlas (TCGA) lung adenocarcinoma and squamous cell lung carcinoma datasets. We functionally validated our findings using adenocarcinoma cell lines and cocultures with peripheral blood mononuclear cells (PBMCs). Our results indicate that growth factor‐dependent MAPK signaling plays an important role in basal and IFNγ‐induced PD‐L1 expression of lung adenocarcinoma without targetable genetic alterations.

## Materials and methods

### TCGA data retrieval and analysis

TCGA RNA sequencing V2 and mutation data for lung adenocarcinoma and squamous cell carcinoma 17, 18 were obtained from the cBioportal for Cancer Genomics 19 on 14 October 2018. We selected 230 adenocarcinoma and 178 squamous cell carcinoma samples with complete RNA sequencing and whole exome sequencing data. Data were analyzed and visualized using R (available from https://www.r‐project.org/) and the R studio interface 1.1.453 (available from https://www.rstudio.com/) and ggplot2 package for R 3.5.1 (available from http://ggplot2.tidyverse.org). Our analysis was performed in 159 lung adenocarcinoma and 166 squamous cell lung carcinoma samples without targetable alterations in *EGFR*, *ALK*, *ROS1*, *BRAF*, *ERBB2*, *MET* or *RET* 20. TCGA RNA sequencing data were normalized in two steps. Each expression value was first log10‐transformed and then Z‐score normalized by subtracting the mean expression of each gene and dividing by the standard deviation. Next, MAPK pathway activation was inferred according to the methods of previously described gene signatures for rat sarcoma (RAS) and MEK activation 21, 22. Inferred pathway activities were calculated as described in the original articles. IFNγ signaling was inferred using *IRF1* and *STAT1* mRNA levels; STAT3 signaling by using *STAT3* gene expression. The correlation of *CD274* (PD‐L1) mRNA level with these signature scores was calculated using Spearman correlation. To complement this analysis, gene set enrichment analysis (GSEA) was performed on the same samples, using the hallmark PI3K and IFNG signatures in addition to the previously mentioned signatures. Furthermore, we used the C6 oncogenic signaling MEK and EGFR signatures and an alternative PI3K signature. However, the authors doubt whether their signatures, developed for estrogen receptor‐positive breast cancer, can be used for other tumor types (http://software.broadinstitute.org/gsea/index.jsp 23, 24). GSEA was performed with 1000 permutations with Z‐score normalized *CD274* gene expression as a continuous phenotype label. Genes were ranked based on the Pearson Metric.

### Cell culture

The human NSCLC cell lines HCC827, H292, A549, H358 and H460 were obtained from the American Type Culture Collection (ATCC, Manassas, VA, USA). H322 was obtained from Sigma‐Aldrich (St Louis, MO, USA). All cell lines are from the adenocarcinoma histological subtype, except H292, which is an adenocarcinoma‐like mucoepidermoid carcinoma. Cells were quarantined until screening for microbial contamination and mycoplasma was performed and proven to be negative. Cells were tested and authenticated using short tandem repeat profiling. Cells were grown in RPMI with 10% FCS, with 2 mm glutamine added for H322 cells. All cells were incubated in a humidified atmosphere with 5% CO_2_ at 37 °C.

### Antibodies and treatments

The details of antibodies used for flow cytometry and Western blotting are provided in Supplementary material, Table S1. Western blotting detection was performed using Lumi‐Light Western blotting substrate (Roche Diagnostics, Basel, Switzerland). Cells were treated under normal culture conditions with EGF, HGF, IFNγ (each from R&D Systems, Minneapolis, MN, USA), erlotinib (LC Laboratories, Woburn, MA, USA), cetuximab (Merck KGaA Darmstadt, Germany), selumetinib (AZD6244, Axon Medchem, Reston, VA, USA), XL147 (LC Laboratories), everolimus (Selleckchem, Houston, TX, USA), BMS911543 (Selleckchem) and actinomycin D (Sigma‐Aldrich).

### siRNA transfection

Cells were transiently transfected with an siRNA targeting *STAT3* (Eurogentec, Liege, Belgium) or a negative control siRNA (12935300, Invitrogen, Carlsbad, CA, USA) using oligofectamine (11252011, Invitrogen) in Opti‐MEM (51985, Invitrogen) according to the manufacturer's instructions. Twenty‐four hours after transfection, cells were treated with indicated ligands and treatments. After a total of 48 h, *STAT3* knockdown efficiency and proteins of interest were analyzed by Western blotting or flow cytometry. All experiments were performed in triplicate.

### Flow cytometric analysis

Cells were harvested using trypsin and kept on ice in PBS with 2% FCS during the entire protocol. After staining, cells were kept in PBS with 2% FCS until analysis. Cells were incubated with anti‐PD‐L1 antibodies at 10 μg/ml for 45 min. Bound antibody was detected by incubating cells with goat anti‐mouse IgG at a 1:50 dilution for 45 min. Measurements were performed on a BD Accuri C6 flow cytometer (BD Biosciences, Franklin Lakes, NJ, USA). Data analysis was performed with FlowJo v10 (Tree Star, Ashland, OR, USA) and surface receptor expression was expressed as mean fluorescence intensity (MFI). Measurements were corrected for background fluorescence and unspecific binding of the secondary antibody. Unless stated otherwise, all experiments were performed in triplicate.

### Western blot

Lysates from cells were made using mammalian protein extraction reagent with protease and phosphatase inhibitors diluted 1:100 (Thermo Fisher Scientific, Waltham, MA, USA). Proteins were separated using SDS‐PAGE. Target proteins were detected with the appropriate antibodies and images were captured using a digital imaging system (Bio‐Rad, Hercules, CA, USA). All bands were observed around the size specified in supplementary material, Table S2A. Densitometric quantitation of target proteins was calculated using ImageJ relative to loading controls β‐actin or GAPDH depending on the target protein size (see supplementary material, Table S2B for data).

### Viability assays

H292 (8000 cells/well), H358 (20 000 cells/well), A549 (2000 cells/well), H322 (10 000 cells/well) and H460 (2000 cells/well) cells were plated in 96‐well plates in their respective media and after 6 h erlotinib or selumetinib was added in concentrations ranging from 0.01 to 10 μm. After 96 h, cells were fixed in 3.7% formaldehyde and stained using crystal violet. After washing, bound crystal violet was dissolved using 10% ethanoic acid and absorption measured at 590 nm. Cell survival was calculated as a percentage of untreated control. All proliferation assays were performed three times in triplicate.

### RNA sample collection and qRT‐PCR

Total RNA was extracted using Trizol reagent (Invitrogen) and possible DNA contamination was removed using TURBO DNase ambion (Life Technologies, AM2238). RNA was then reverse transcribed to cDNA with M‐MLV reverse transcriptase (Thermo Fisher Scientific, 28025013). Real‐time PCR was performed using IQ SYBR Green Supermix (Bio‐Rad, 1708886) according to the manufacturer's instructions. The following primers were used: *CD274* forward 5′‐CAATGTGACCAGCACACTGAGAA‐3′, reverse 5′‐GGCATAATAAGATGGCTCCCAGAA‐3′; *GAPDH* forward 5′‐CCCACTCCTCCACCTTTGAC‐3′, reverse 5′‐CCACCACCCTGTTGCTGTAG‐3′. The relative gene expression was calculated using the double delta CT method and *GAPDH* as a loading control 25. All qPCR experiments were performed three times in duplicate.

### Coculture experiments

Human PBMCs were isolated from whole blood by Ficoll‐Paque density centrifugation (Ficoll‐Paque PLUS, GE Healthcare Life Sciences, Marlborough, MA, USA) from peripheral blood donated by healthy volunteers. The acquired PBMCs were activated for 72 h using human T‐activator CD3/28 beads (Thermo Fisher Scientific) and 100 IU/ml IL‐2 (Proleukin, Novartis, Basel, Switzerland) in the presence of tumor cells. Separately, tumor cells were seeded into 96‐well plates at a density of 1 × 10^4^ cells/well for 48 h. Then, the pre‐activated PBMCs were added into the coculture system at a 5:1 ratio of PBMCs to tumor cells. During coculture, cells were treated with EGF (20 ng/ml), erlotinib (10 μm) and selumetinib (10 μm). After 24 h of coculture, cell‐free supernatant was collected for IFNγ analysis by ELISA (Sino Biological, Beijing, PR China). Cells were harvested for flow cytometric measurement of membrane PD‐L1. In separate experiments, tumor cells were cultured in cell‐free supernatant from activated PBMCs. Membrane PD‐L1 levels were determined after 24 h using flow cytometry.

### Statistics

Cell line experiments were assessed for differences with unpaired two‐tailed Student's *t*‐test or two‐way ANOVA followed by Bonferroni post‐hoc or Dunnett's test. Results are represented as means ± SD. A *P* value < 0.05 was considered statistically significant. Statistical analyses were performed using GraphPad Prism software (version 6.0 GraphPad software).

## Results

### MAPK pathway activation correlates with *CD274* gene expression in lung adenocarcinoma

**Figure 1:**
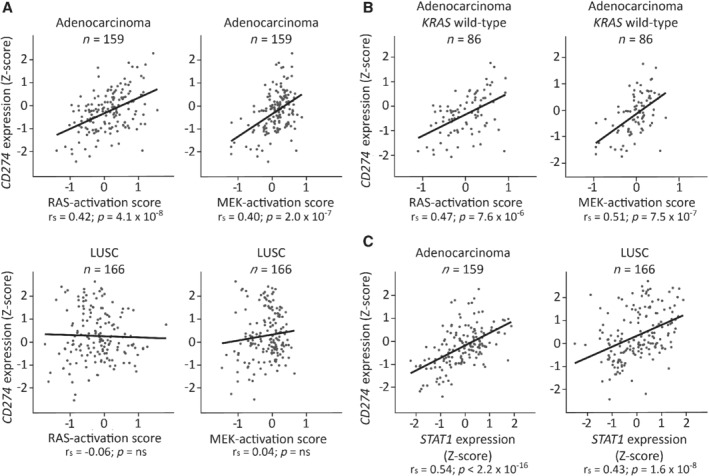
MAPK and IFNγ signaling correlate with *CD274* expression in lung adenocarcinoma tumors without targetable genetic alterations. RNA sequencing data from all TCGA lung adenocarcinoma and squamous cell lung carcinoma samples without targetable genetic alterations were collected. RNA sequencing data were Z‐score normalized after ^10^log transformation. (A) Spearman correlation between *CD274* and RAS activation score or MEK activation score in all samples. (B) Spearman correlation between *CD274* expression and RAS activation score or MEK activation score in *KRAS* wild‐type lung adenocarcinoma samples. (C) Spearman correlation between *STAT1* and *CD274* in all samples. LUSC, squamous cell lung carcinoma.

To study which EGFR‐related signaling pathways regulate *CD274* expression in NSCLC without targetable genetic alterations, we collected RNA sequencing data of 159 lung adenocarcinoma and 166 squamous cell lung carcinoma samples from TCGA, excluding samples with driver mutations in *EGFR*, *ALK*, *BRAF*, *ROS1*, *MET*, *ERBB2* and *RET*. Activating *KRAS* mutations were present in 75 of the lung adenocarcinoma and in one of the squamous cell carcinoma samples. Activation of the MAPK pathway was determined using validated signatures for RAS or MEK activation 21, 22. MAPK pathway activation scores were significantly higher in *KRAS* mutant samples (see supplementary material, Figure S1A). In addition, there was a moderate correlation between RAS and MEK activation scores (see supplementary material, Figure S1B). Interestingly, in adenocarcinomas, but not in squamous cell carcinomas, RAS and MEK activation scores correlated with *CD274* gene expression (Figure 1A, Table 1). Subset analysis showed that these correlations were strongest in *KRAS* wild‐type lung adenocarcinomas (Figure 1B and supplementary material, Figure S1C). *STAT3* mRNA levels did not correlate with *CD274* in any subset (Table 1). In both histological subtypes, mRNA levels of *STAT1* and *IRF1*, important mediators of IFNγ, correlated with *CD274* (Figure 1C and supplementary material, Figure S1D), *CD8A* (r_s_ = 0.73, r_s_ = 0.72), and *IFNG* (r_s_ = 0.71, r_s_ = 0.74), respectively. Inferred MEK and RAS activities were not significantly correlated with *STAT1* (r_s_ = 0.12, *p* = 0.27; r_s_ = 0.1, *p* = 0.36). Interestingly, a combined score for MAPK and IFNγ signaling, created by adding up 0 to 1 rescaled MEK activation scores and *STAT1* levels, correlated more strongly with *CD274* mRNA levels (see supplementary material, Figure S1E). GSEA using RAS, MEK and the hallmark IFNG signatures largely confirmed these findings. In addition, GSEA of the PI3K hallmark signature suggested a link between PI3K/mTOR signaling and *CD274* (see supplementary material, Table S3). This suggests that activation of the MAPK, PI3K/mTOR and IFNγ pathways is related to increased *CD274* mRNA levels in lung adenocarcinomas without targetable genetic alterations.

**Table 1:** Correlation between inferred MAPK, PI3K/mTOR and IFNγ pathway activity, and *CD274* gene expression in NSCLC subtypes

		Adenocarcinoma				LUSC
		Non‐targetable				
		Total *n* = 159	*KRAS*mt *n* = 73	*KRAS*wt *n* = 86	*EGFR*mt Total *n* = 28	Non‐targetable *KRAS*wt *n* = 166
RAS score	r_s_	0.42	0.32	0.47	0.52	−0.06
*p*	4.1 × 10^−8^	0.006	7.6 × 10^−6^	0.005	ns
MEK score	r_s_	0.40	0.21	0.51	0.13	0.04
*p*	2.0 × 10^−7^	ns	7.5 × 10^−7^	ns	ns
*STAT3*	r_s_	−0.15	−0.20	−0.10	−0.08	0.11
*p*	ns	ns	ns	ns	ns
*STAT1*	r	0.54	0.66	0.48	0.33	0.43
*p*	< 2.2 × 10^−16^	< 2.2 × 10^−16^	4.9 × 10^−6^	ns	1.6 × 10^−8^
*IRF1*	r	0.49	0.43	0.53	0.47	0.33
*p*	8.8 × 10^−11^	2.0 × 10^−4^	2.0 × 10^−7^	0.01	2.1 × 10^−5^

mt, mutant; ns, not significant; r_s,_ Spearman's rho; wt, wild‐type.

### EGF increases IFNγ‐induced PD‐L1 expression in NSCLC cells without targetable genetic alterations

**Figure 2:**
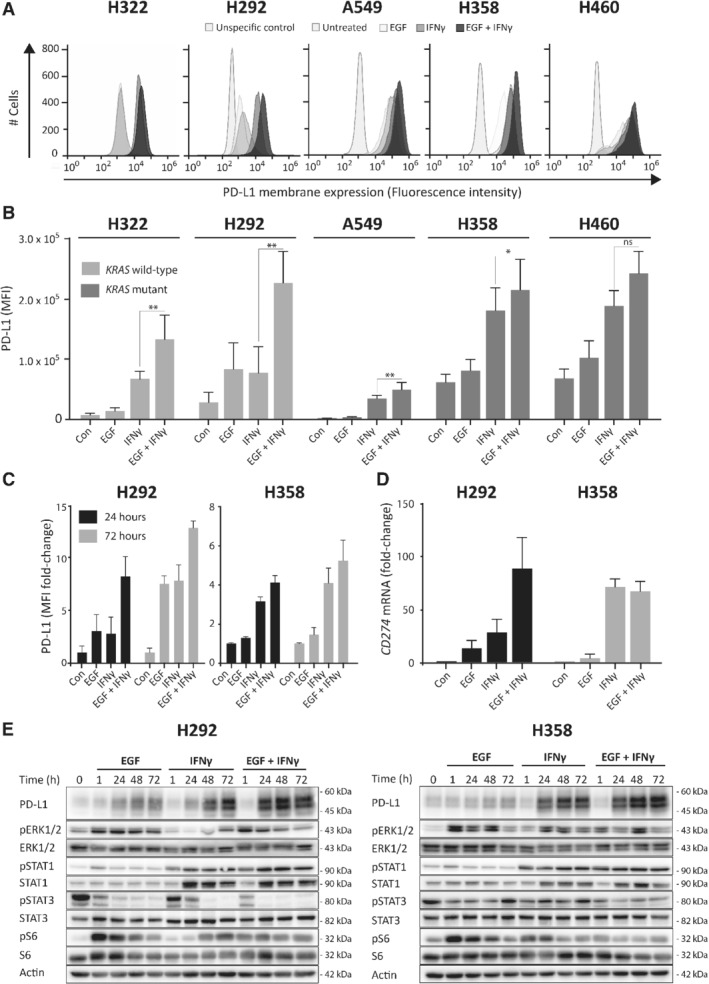
EGF and IFNγ induce PD‐L1 in NSCLC cell lines. (A and B) NSCLC cell lines without targetable genetic alterations treated with 20 ng/ml EGF, 20 ng/ml IFNγ or both. After 24 h, PD‐L1 membrane expression was measured using flow cytometry (Student's *t*‐test; ns = not significant, *p < 0.05, **p < 0.01 compared with IFNγ, *n* = 3–11, combined data from all other figures). (C) Membrane PD‐L1 levels measured using flow cytometry in cells treated with EGF and IFNγ for 24 or 72 h. Data are represented as MFI/mean corresponding control MFI, *n* = 3. (D) PD‐L1 mRNA levels measured using RT‐qPCR on H292 and H358 treated with 20 ng/ml EGF or IFNγ or both for 24 h. Data were analyzed using the double delta CT method and *GAPDH* as a loading control. (E) Western blotting of protein extracts from H292 and H358 treated with 20 ng/ml EGF, 20 ng/ml IFNγ or both for 1, 24, 48 and 72 h. Actin was used as a loading control. Con, untreated control.

A panel of lung adenocarcinoma cell lines without targetable genetic alterations, including a *KRAS* wild‐type (H322), three *KRAS* mutant (A549, H358 and H460) and a *KRAS* wild‐type adenocarcinoma‐like mucoepidermoid carcinoma cell line (H292) 26, was selected to further investigate the relationship between EGFR and IFNγ pathway activation with PD‐L1 expression. Membrane PD‐L1 was observed in all cell lines, irrespective of *KRAS* mutation status (see supplementary material, Figure S2A). The highest levels were found in H292, H358 and H460 cells. Levels were comparable with PD‐L1 membrane levels of HCC827 *EGFR* mutant NSCLC cells (see supplementary material, Figure S2A). We wondered whether IFNγ and EGF, known activators of the EGFR, PI3K/mTOR and MAPK pathways, would increase PD‐L1 levels in our panel. Treatment with EGF or IFNγ for 24 h using physiologically relevant concentrations (20 ng/ml) 27, 28 increased membrane PD‐L1 in both *KRAS* wild‐type and *KRAS* mutant cells (Figure 2A,B). Moreover, exposure of cells to EGF combined with IFNγ resulted in a further increase in PD‐L1 compared with IFNγ alone. Prolonged incubation up to 72 h further enhanced PD‐L1 in H292 and H358 (Figure 2C).

To gain insight into the underlying mechanism of the increase in surface PD‐L1, we measured total PD‐L1 protein and *CD274* mRNA levels. The EGF‐ and IFNγ‐induced increase in surface expression was reflected in a strong induction of both mRNA and total protein levels (Figure 2D,E). EGF stimulated the MAPK and PI3K/mTOR pathways, as signified by increased levels of phosphorylated ERK 1 and 2 (pERK1/2) and phosphorylated ribosomal S6 protein (pS6), respectively (Figure 2E and supplementary material, Figure S2B). IFNγ strongly increased STAT1 and phosphorylated STAT1 (pSTAT1) levels for up to 72 h. Taken together, these results indicate that EGF and IFNγ activate the MAPK, PI3K/mTOR and STAT1 pathways, and concurrently increase PD‐L1 mRNA, protein and membrane levels.

### EGFR inhibition prevents EGF‐ and IFNγ‐induced PD‐L1 upregulation

**Figure 3:**
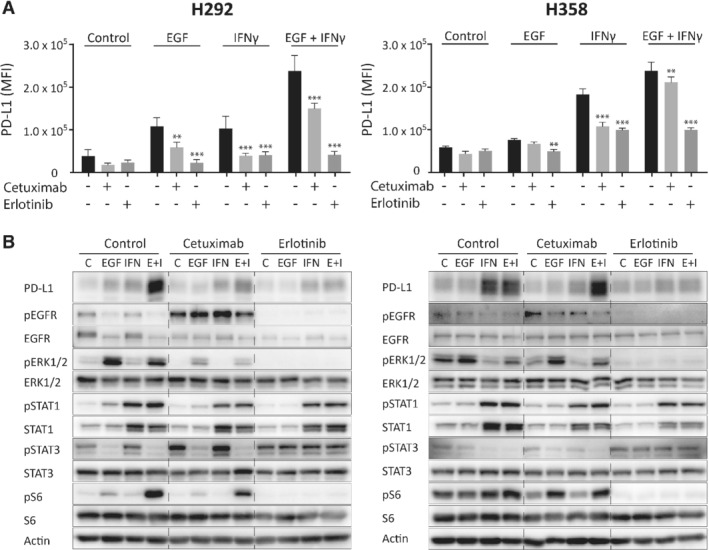
EGFR inhibition prevents EGF‐ and IFNγ‐induced PD‐L1 expression. H292 and H358 cells were treated with 20 μg/ml cetuximab or 10 μm erlotinib with and without cotreatment with 20 ng/ml EGF, 20 ng/ml IFNγ or both for 24 h. (A) Membrane PD‐L1 measured using flow cytometry (two‐way ANOVA with Dunnett's multiple comparisons test; **p < 0.01, ***p < 0.001 compared with untreated control). (B) Cellular protein levels measured using Western blotting. Actin was used as a loading control. Data from a representative experiment are shown. C, untreated control; E + I, EGF + IFNγ.

To analyze the regulation of membrane PD‐L1 by EGFR and STAT1 signaling, H292 and H358 cells were treated with EGF and IFNγ in the presence of anti‐EGFR monoclonal antibody cetuximab or EGFR small molecule inhibitor erlotinib. Interestingly, cetuximab and erlotinib prevented not only EGF‐induced but also IFNγ‐induced upregulation of *CD274* mRNA levels, which was reflected in reduced membrane and total PD‐L1 protein levels (Figure 3A,B and supplementary material, Figure S3A). Data from multiple experiments showed that erlotinib reduced basal PD‐L1 membrane levels in H358 but not H292 cells (see supplementary material, Figure S3B). Erlotinib had a similar effect on EGF‐ and IFNγ‐induced PD‐L1 membrane levels in two other cell lines but not in the erlotinib‐resistant H460 cell line, as expected (see supplementary material, Figure S4A). EGFR inhibition effectively reduced EGF‐dependent MAPK and PI3K/mTOR signaling and modestly decreased IFNγ‐induced upregulation of (p)STAT1 (Figure 3B). These results indicate that EGF‐ and IFNγ‐induced *CD274* mRNA and PD‐L1 protein and membrane levels are dependent on EGFR‐mediated signaling.

### MAPK pathway inhibition prevents PD‐L1 induction by EGF and IFNγ

**Figure 4:**
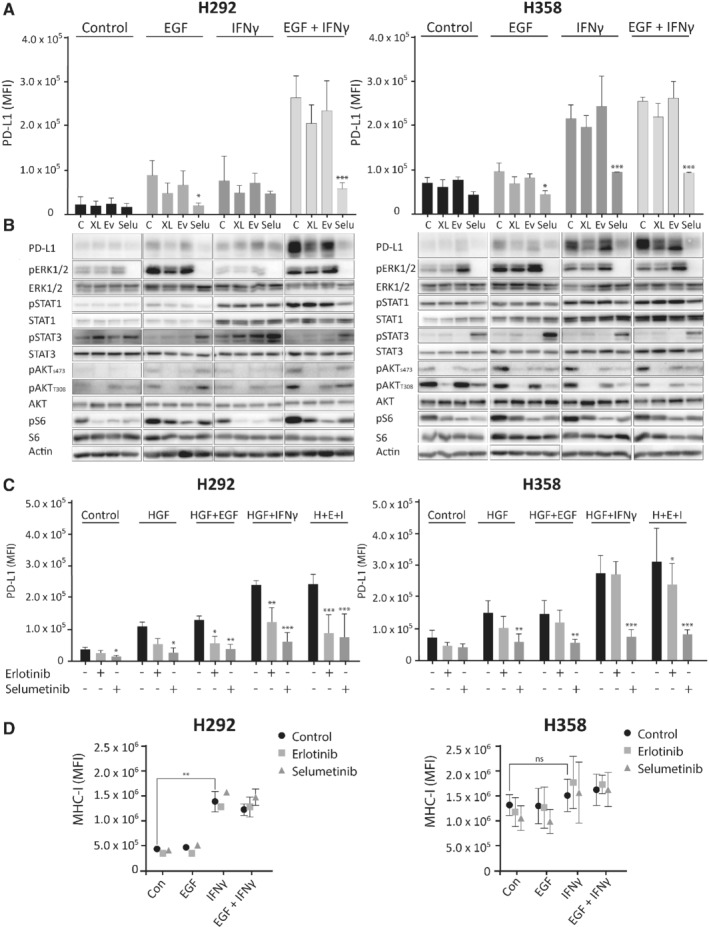
Selumetinib effectively decreases growth factor‐ and IFNγ‐induced PD‐L1 expression. H292 and H358 cells were treated with 10 μm XL147, 10 μm everolimus or 10 μm selumetinib, with and without cotreatment with 20 ng/ml EGF, 20 ng/ml IFNγ or both for 24 h. (A) Flow cytometry of membrane PD‐L1 (two‐way ANOVA with Dunnett's multiple comparisons test, *p < 0.05, ***p < 0.001 compared with ligand‐stimulated control). (B) Western blot of cellular protein levels. Actin was used as a loading control. Data are from a representative experiment (*n* = 2). (C) Membrane PD‐L1 and (D) MHC‐1 levels measured by flow cytometry of H292 and H358 cells treated with 10 μm erlotinib or 10 μm selumetinib, with and without cotreatment with 20 ng/ml EGF, 20 ng/ml HGF, 20 ng/ml IFNγ or a combination for 24 h. In (C), two‐way ANOVA with Dunnett's multiple comparisons test, *p < 0.05, **p < 0.01, ***p < 0.001 compared with ligand‐stimulated control. (D) Student's *t*‐test, **p < 0.01. ns, not significant; C, untreated control; XL, XL147; Ev, everolimus; Selu, selumetinib. H + E + I, HGF + EGF + IFNγ.

Next, we assessed the involvement of MAPK and PI3K/mTOR signaling in PD‐L1 upregulation. Selumetinib, an inhibitor of MEK1/2, almost completely suppressed induction of *CD274* mRNA by EGF and IFNγ in H292 and H358, and diminished the induction of protein and membrane levels (Figure 4A,B and supplementary material, Figure S3A). Moreover, selumetinib decreased basal PD‐L1 membrane levels in H292 and H358 cells (see supplementary material, Figure S3B). Selumetinib effectively inhibited MEK1/2 activity, as reflected in the reduction in pERK1/2 levels, and had a moderate effect on pSTAT1 levels. The effect of selumetinib on membrane PD‐L1 was confirmed in additional cell lines (see supplementary material, Figure S4A). PI3K inhibitor XL147 and mTORC1 inhibitor everolimus partially suppressed EGF‐ and IFNγ‐induced PD‐L1 in H292 cells, but they had no effect on the induction of membrane PD‐L1 (Figure 4A,B). At these concentrations, both drugs effectively inhibited PI3K/mTOR pathway activity, as indicated by reduced pAKT and pS6 levels (Figure 4B).

We pursued studying the MAPK pathway because of its major influence on PD‐L1 expression. A wide range of selumetinib and erlotinib concentrations was used to determine if lower drug concentrations also reduce PD‐L1 expression. After 24 h, even the lowest selumetinib concentration (0.1 μm) strongly reduced pERK levels, as well as PD‐L1 protein and membrane expression levels of H292 and H358 cells in both control and EGF‐ and IFNγ‐stimulated cells (see supplementary material, Figure S3C). Treatment with this selumetinib concentration for 96 h resulted in a growth reduction of 30–50% (see supplementary material, Figure S3D), indicating that PD‐L1 expression can be manipulated with a MAPK activity inhibitor using concentrations that only partially reduce cell growth. In line with these results, selumetinib had a similar moderate effect on growth in the other three cell lines (see supplementary material, Figure S4B). Similar results were observed with erlotinib. In conclusion, MAPK pathway inhibitors suppress EGF‐ and IFNγ‐induced *CD274* mRNA and PD‐L1 protein and membrane expression at concentrations that have a small effect on growth.

### HGF induces surface PD‐L1 via the MAPK pathway

We investigated whether activation of the MAPK pathway via HGF receptor (cMET) has a similar effect on PD‐L1 as MAPK activation by EGFR. Overexpression of cMET and its ligand HGF occur frequently in lung adenocarcinoma tumors 29. Moreover, upon binding of HGF, cMET is known to activate PI3K/mTOR, MAPK and JAK/STAT pathways, similar to EGFR 30. HGF enhanced PD‐L1 expression and augmented IFNγ‐induced PD‐L1 expression in the cMET‐positive H292 and H358 cell lines (Figure 4C). Combining HGF and EGF had no additional effect on membrane PD‐L1 levels compared with single EGF or HGF treatment. Also, in this case, selumetinib effectively prevented HGF‐induced effects on PD‐L1 levels in both cell lines, whereas erlotinib only showed efficacy in H292 cells. Taken together, these results demonstrate that, irrespective of the upstream growth factor receptor, MAPK pathway activation is essential for PD‐L1 membrane expression.

### MAPK pathway inhibition does not interfere with IFNγ‐induced MHC‐I upregulation

Expression of MHC‐I is critical for tumor cell antigen presentation and the anti‐tumoral immune response 31. Because both MAPK and IFNγ signaling can influence MHC‐I expression, we wondered whether EGFR and MEK1/2 blockade could interfere with its expression in our cell lines 32, 33, 34. IFNγ, but not EGF, increased MHC‐I membrane expression in four of five cell lines (Figure 4D and supplementary material, Figure S4C). Moreover, MAPK pathway inhibition using erlotinib and selumetinib did not influence IFNγ‐induced upregulation of MHC‐I expression, suggesting that MHC‐I‐mediated tumor cell antigen presentation will not be impaired by these drugs.

### MAPK signaling increases stability of *CD274* mRNA

**Figure 5:**
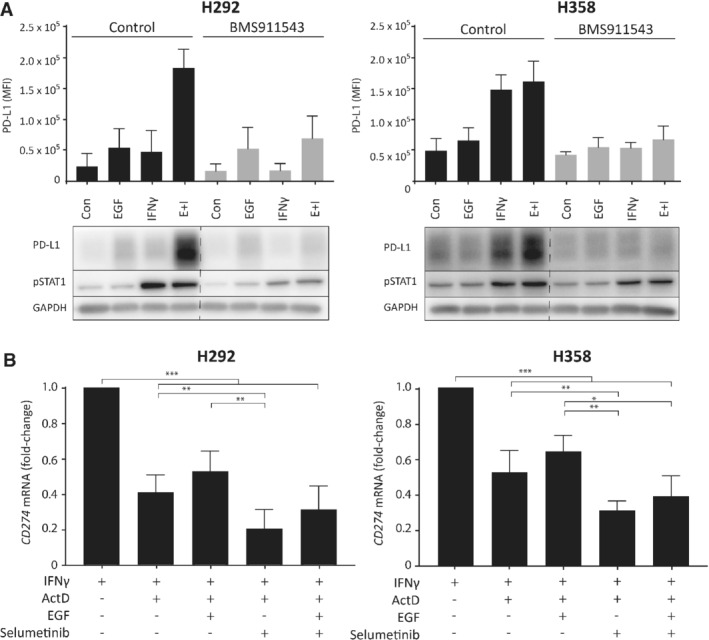
MAPK signaling increases *CD274* mRNA stability. (A) Membrane PD‐L1 using flow cytometry and cellular protein levels by Western blotting in cells treated with BMS911543 in the presence of 20 ng/ml EGF and IFNγ for 24 h. Blot from a representative experiment (*n* = 2). (B) RT‐qPCR of *CD274* mRNA levels (PD‐L1) in H292 and H358 cells initially treated with IFNγ for 24 h, then with 5 μg/ml actinomycin D for 10 min, followed by 20 ng/ml IFNγ, 20 ng/ml EGF or 10 μm selumetinib for 80 min. Data were analyzed using the double delta CT method and *GAPDH* as a loading control (two‐way ANOVA with Tukey's test, *p < 0.05, **p < 0.01, ***p < 0.001). Con, untreated control; E + I, EGF + IFNγ; ActD, actinomycin D.

To investigate the role of STAT signaling in MHC‐I and PD‐L1 expression after IFNγ or EGF treatment, we inhibited STAT1 and STAT3, major transcription factors downstream of IFNγ and EGFR signaling, respectively 6, 35. Suppression of STAT1 signaling using JAK2 inhibitor BMS911543 prevented IFNγ‐induced PD‐L1 and MHC‐I expression, but not EGF‐induced PD‐L1 expression (Figure 5A and supplementary material, Figure S5A). Also, inhibition of STAT3 using an siRNA had no influence on PD‐L1 regulation by MAPK signaling (see supplementary material, Figure S5B). Therefore, we hypothesized that the MAPK pathway may regulate PD‐L1 at a post‐transcriptional level. *KRAS* mutations were recently shown to be involved in post‐transcriptional regulation of basal PD‐L1 levels through modulation of *CD274* mRNA stability 36. To study whether MAPK signaling controls the stability of IFNγ‐induced *CD274* mRNA, *KRAS* wild‐type and mutant cells were pretreated with IFNγ followed by the addition of the transcriptional blocker actinomycin D 37. Blocking transcription for 90 min halved *CD274* levels (Figure 5B). Interestingly, degradation of *CD274* was counteracted by EGF‐induced activation of MAPK signaling. Accordingly, inhibition of MAPK signaling with selumetinib accelerated *CD274* degradation and decreased the stabilization by EGF. These results show that MAPK signaling influences the stability of *CD274* mRNA, contributing to regulation of PD‐L1 protein and membrane expression.

### MAPK pathway inhibition decreases PBMC‐induced PD‐L1 surface expression

**Figure 6:**
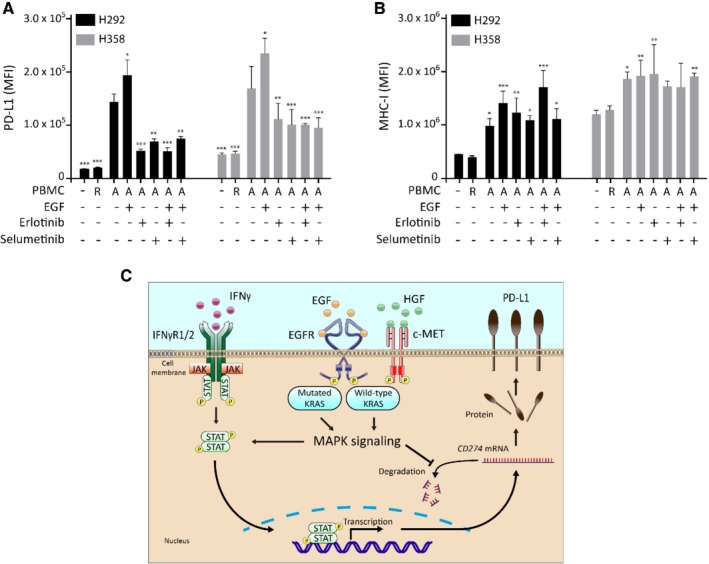
Erlotinib and selumetinib prevent PBMC‐induced PD‐L1, but not MHC‐I expression, in NSCLC cells. (A) H292 and H358 cells were cocultured with 72 h pre‐activated PBMCs from healthy volunteers at a ratio of five PBMCs per tumor cell. During coculture, cells were treated with 20 ng/ml EGF, and 10 μm erlotinib or 10 μm selumetinib. Membrane PD‐L1 was measured by flow cytometry after 24 h (two‐way ANOVA with Bonferroni's multiple comparisons method, **p < 0.01, ***p < 0.01 compared with control + pre‐activated PBMCs). (B) Membrane MHC‐I measured using flow cytometry (two‐way ANOVA, *p < 0.05, **p < 0.01 compared with control). R, resting PBMCs; A, activated PBMCs. (C) Proposed model for the role of IFNγ and MAPK signaling in PD‐L1 regulation in lung adenocarcinoma. IFNγ derived from tumor infiltrating immune cells induces *CD274* transcription in tumor cells through activation of JAK/STAT signaling. *CD274* mRNA is translated into PD‐L1 protein, which is transported to the cell membrane. Growth receptor‐ and mutated KRAS‐induced MAPK signaling increases STAT signaling, potentially adding to transcriptional activity. Also, MAPK signaling increases the stability of *CD274* mRNA, resulting in increased mRNA and protein levels, and subsequently increasing PD‐L1 membrane expression.

To study the relationship between immune cell activation and PD‐L1 expression of tumor cells, we performed cocultures of PBMCs and NSCLC cells. After 24 h coculture, membrane PD‐L1 and MHC‐I were strongly induced in tumor cells (Figure 6A,B). Conditioned medium from activated PBMCs contained IFNγ (30 ng/ml) in comparable levels to our other experiments and also strongly induced membrane PD‐L1, indicating that IFNγ may be involved in PBMC‐induced PD‐L1 (see supplementary material, Figure S6A,B). Similar to our previous experiments, EGF further enhanced tumor cell MAPK pathway activity and PD‐L1 expression, which was counteracted by erlotinib and selumetinib, without influencing MHC‐I (Figure 6A,B and supplementary material, Figure S6A). Our results indicate that MAPK pathway inhibition can reduce tumor cell PD‐L1 in a more complex coculture system, without interfering with MHC‐I induction in tumor cells, potentially improving the immunogenicity of these cells.

## Discussion

In this study we revealed a correlation between MAPK pathway activation and *CD274* expression in lung adenocarcinomas without targetable genetic alterations using TCGA RNA sequencing data. Subsequently, we demonstrated the importance of MAPK signaling in the upregulation of PD‐L1 by growth factors and IFNγ in lung adenocarcinoma cell lines, which was mediated through *CD274* mRNA stability and STAT1 activation (Figure 6C). Inhibition of the MAPK pathway prevents growth factor‐, IFNγ‐ and PBMC‐induced PD‐L1 upregulation, whereas it does not interfere with MHC‐I expression. Taken together, these results indicate that MAPK pathway inhibition may improve tumor cell immunogenicity of lung adenocarcinomas without targetable genetic alterations, comprising almost 60% of all lung adenocarcinoma tumors in the Western world 3.

Our TCGA analysis suggests that MAPK pathway activity and *CD274* gene expression are primarily connected in lung adenocarcinoma, but not in squamous cell carcinoma of the lung. Targeting the MAPK pathway is especially interesting in lung adenocarcinoma, because these tumors have a more active MAPK pathway and more frequently harbor *KRAS* mutations compared with squamous cell carcinoma tumors 38, 39. Intriguingly, this is also the subtype where tumor cell PD‐L1 has predictive and prognostic value 40, 41. Our study shows that MAPK pathway inhibition prevents the induction of *CD274* mRNA by EGF and IFNγ through two separate mechanisms (Figure 6C). First, a moderate dose‐dependent reduction of STAT1 and pSTAT1 levels upon inhibition of EGFR or MEK1/2 may lower *CD274* transcription (see supplementary material, Figure S3C). This might be due to inhibition of the eukaryotic initiation factor 4F (eIF4F) translation initiation complex, which is a downstream effector of the MAPK pathway and essential for cap‐dependent translation of *STAT1* mRNA 39, 42. Second, we observed reduced *CD274* mRNA stability upon inhibition of MEK1/2 or EGFR. This expands earlier data on basal PD‐L1 expression in *KRAS* mutant cell lines, where MEK1/2 inhibition activates tristetraprolin, resulting in *CD274* mRNA degradation 36. Although *CD274* transcription and MHC‐I‐related transcription are both regulated by STAT1 6, 35, MAPK inhibition, in contrast to JAK2 inhibition, does not affect MHC‐I expression, suggesting that MAPK activity primarily regulates *CD274* mRNA stability in lung adenocarcinoma cells. At the protein level, PD‐L1 can be affected by several mechanisms, such as glycosylation, ubiquitination and stabilization at the cell membrane 15, 43, 44, 45. However, we found no direct effect of EGF or IFNγ on CKLF‐like MARVEL transmembrane domain containing protein 6 (CMTM6) protein levels in H292 or H358 cells (data not shown).

Our experiments demonstrated that both EGFR and MEK1/2 inhibitors decrease EGF‐ and IFNγ‐induced PD‐L1 expression, potentially increasing immunogenicity of lung adenocarcinoma cells. Nevertheless, because the MAPK pathway is downstream of a plethora of growth factor receptors, downstream inhibition with MEK1/2 inhibitors may be more effective to modulate PD‐L1 than inhibition of specific growth factor receptors. This is supported by our finding that MEK1/2 inhibition, but not EGFR inhibition, prevented HGF‐induced PD‐L1 expression and by earlier findings in renal cell carcinoma 46. *In vitro*, we observed PD‐L1 downregulation at selumetinib concentrations that had a small effect on cell growth. Although *in vitro* experiments do not perfectly model the tumor microenvironment, our results suggest that an immunomodulatory effect may already be present at lower selumetinib doses than previously utilized in NSCLC patients 47, 48. The immunomodulatory role of MAPK signaling is increasingly being recognized 49. Multiple studies using *in vivo* colon cancer models showed that MEK inhibition potentiates the anti‐tumor immune response by preventing T cell apoptosis and decreasing levels of myeloid suppressor cells and regulatory T cells. This resulted in sustained tumor regression when combined with PD‐L1, PD‐1 or CTLA‐4 blocking treatment 50, 51, 52. In phase II and III studies of selumetinib in NSCLC patients, disappointing efficacy was observed 47, 48. However, clear immune modulating effects were observed, indicating that MAPK pathway inhibition may increase the efficacy of immunotherapy. Modulating PD‐L1 expression is especially interesting in NSCLC tumors without targetable genetic alterations, because these have a more inflamed tumor microenvironment and respond better to immune checkpoint inhibitors than tumors with targetable genetic alterations, such as *EGFR* mutations 1, 2. These combination strategies are currently being tested in NSCLC patients (NCT03600701, NCT03299088).

In conclusion, our results show the importance of growth factor‐induced MAPK pathway signaling in PD‐L1 expression in lung adenocarcinoma without targetable genetic alterations. This provides a rationale to explore the combination of MAPK pathway inhibitors with immunotherapy in this lung cancer subtype.

## Author contributions statement

TS, AK, AT and SJ designed, performed and interpreted the experiments. EV gave valuable input on structuring the experiments. MB and RF, respectively, guided the coculture and *in silico* experiments. TS and AK wrote the manuscript and put the figures together. EV, MB, RF, AT and SJ made revisions and proofread the manuscript. All authors read and approved the final manuscript.

**SUPPLEMENTARY MATERIAL ONLINE**

**Figure S1.** Supplementary TCGA analyses

**Figure S2.** EGF and IFNγ increase PD‐L1 expression in lung adenocarcinoma cell lines without targetable genetic alterations

**Figure S3.** MAPK pathway inhibition decreases EGF‐ and IFNγ‐induced PD‐L1 expression at concentrations that only partially reduce cell growth

**Figure S4.** Effects of erlotinib and selumetinib on PD‐L1 and MHC‐I expression and proliferation in a panel of NSCLC cell lines

**Figure S5.** JAK2 and STAT3 influence MHC‐I, but not PD‐L1 signaling

**Figure S6.** PBMC‐derived IFNγ may play a role in PD‐L1 induction

**Table S1.** Antibodies used in the study

**Table S2.** (A) Protein band sizes that were considered as specific staining for the target protein. (B) Quantitation of densitometry of all presented Western blotting results

**Table S3.** Gene set enrichment results

## Supporting information


**Figure S1.** Supplementary TCGA analyses

**Figure S2.** EGF and IFNγ increase PD‐L1 expression in lung adenocarcinoma cell lines without targetable genetic alterations

**Figure S3.** MAPK pathway inhibition decreases EGF‐ and IFNγ‐induced PD‐L1 expression at concentrations that only partially reduce cell growth

**Figure S4.** Effects of erlotinib and selumetinib on PD‐L1 and MHC‐I expression and proliferation in a panel of NSCLC cell lines

**Figure S5.** JAK2 and STAT3 influence MHC‐I, but not PD‐L1 signaling

**Figure S6.** PBMC‐derived IFNγ may play a role in PD‐L1 induction

**Table S1.** Antibodies used in the study

**Table S2.** (A) Protein band sizes that were considered as specific staining for the target protein. (B) Quantitation of densitometry of all presented Western blotting results

**Table S3.** Gene set enrichment results
