# Supplementary figures and images for: MAPK pathway activity plays a key role in PD‐L1 expression of lung adenocarcinoma cells
Source: J Pathol. 2019 May 21;249(1):52–64. doi: 10.1002/path.5280 (PMC6767771; doi:10.1002/path.5280)

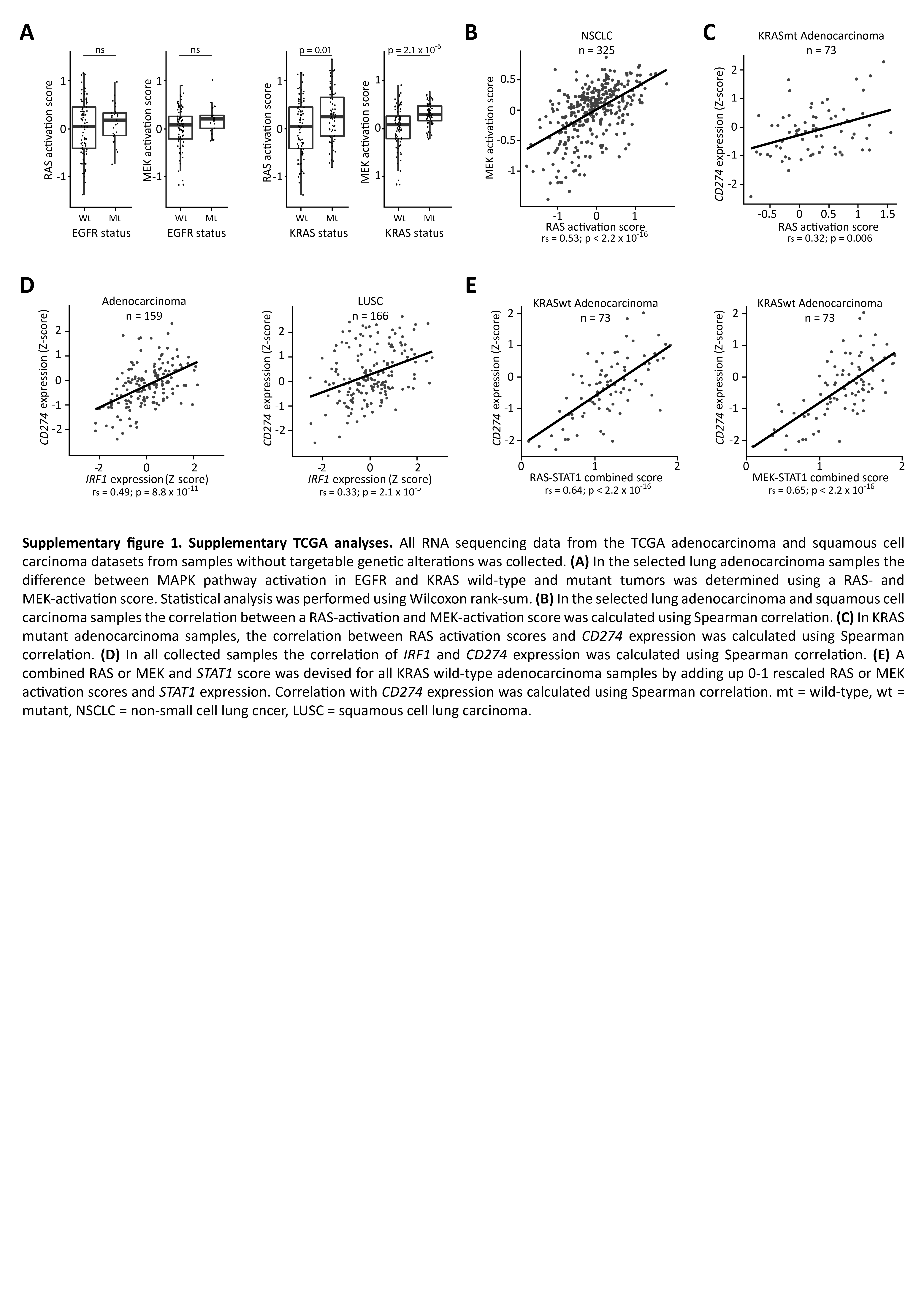

Supplement: Supplementary file 1 — Figure S1. Supplementary TCGA analyses [file PATH-249-52-s001.tif]

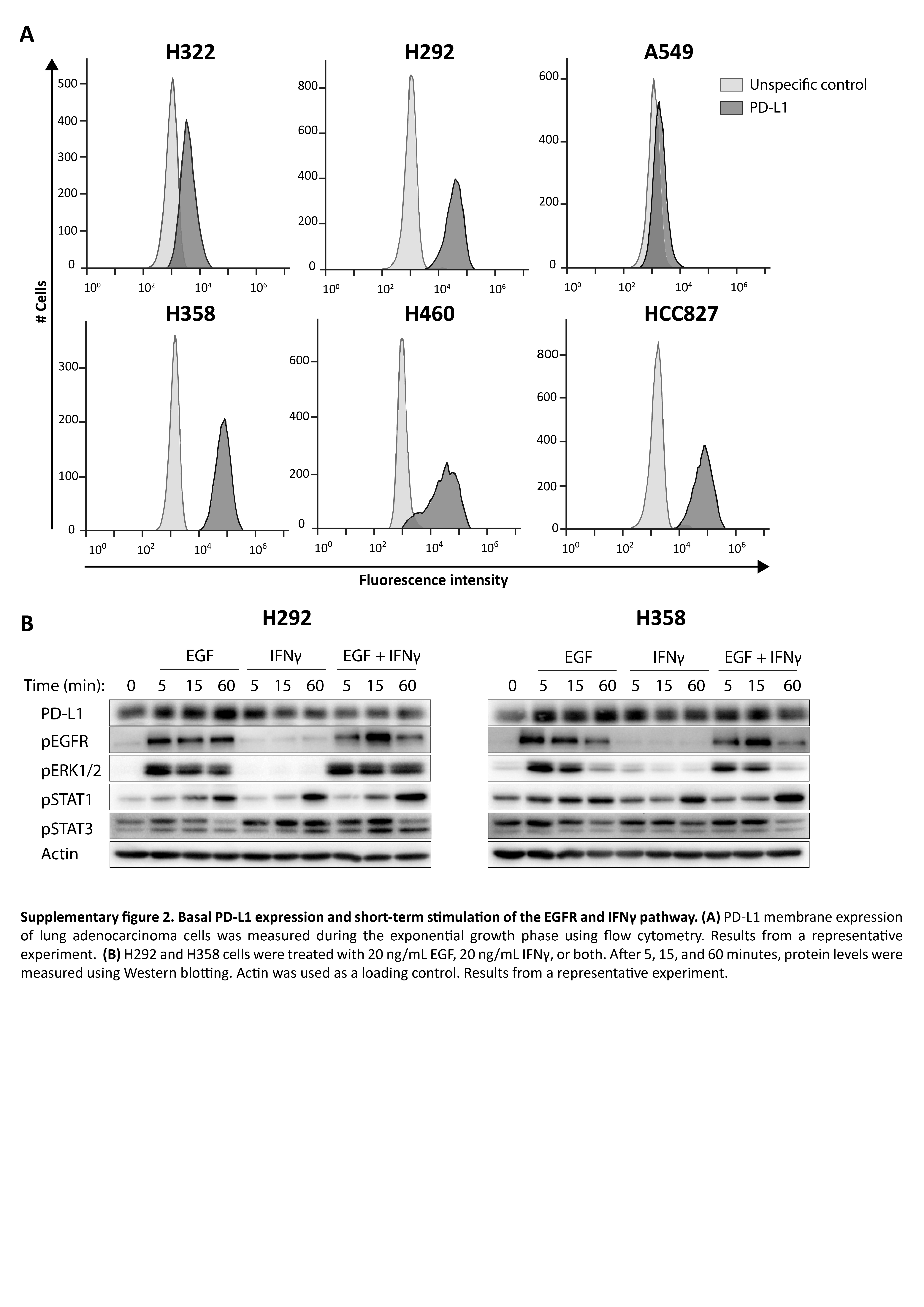

Supplement: Supplementary file 2 — Figure S2. EGF and IFNγ increase PD‐L1 expression in lung adenocarcinoma cell lines without targetable genetic alterations [file PATH-249-52-s002.tif]

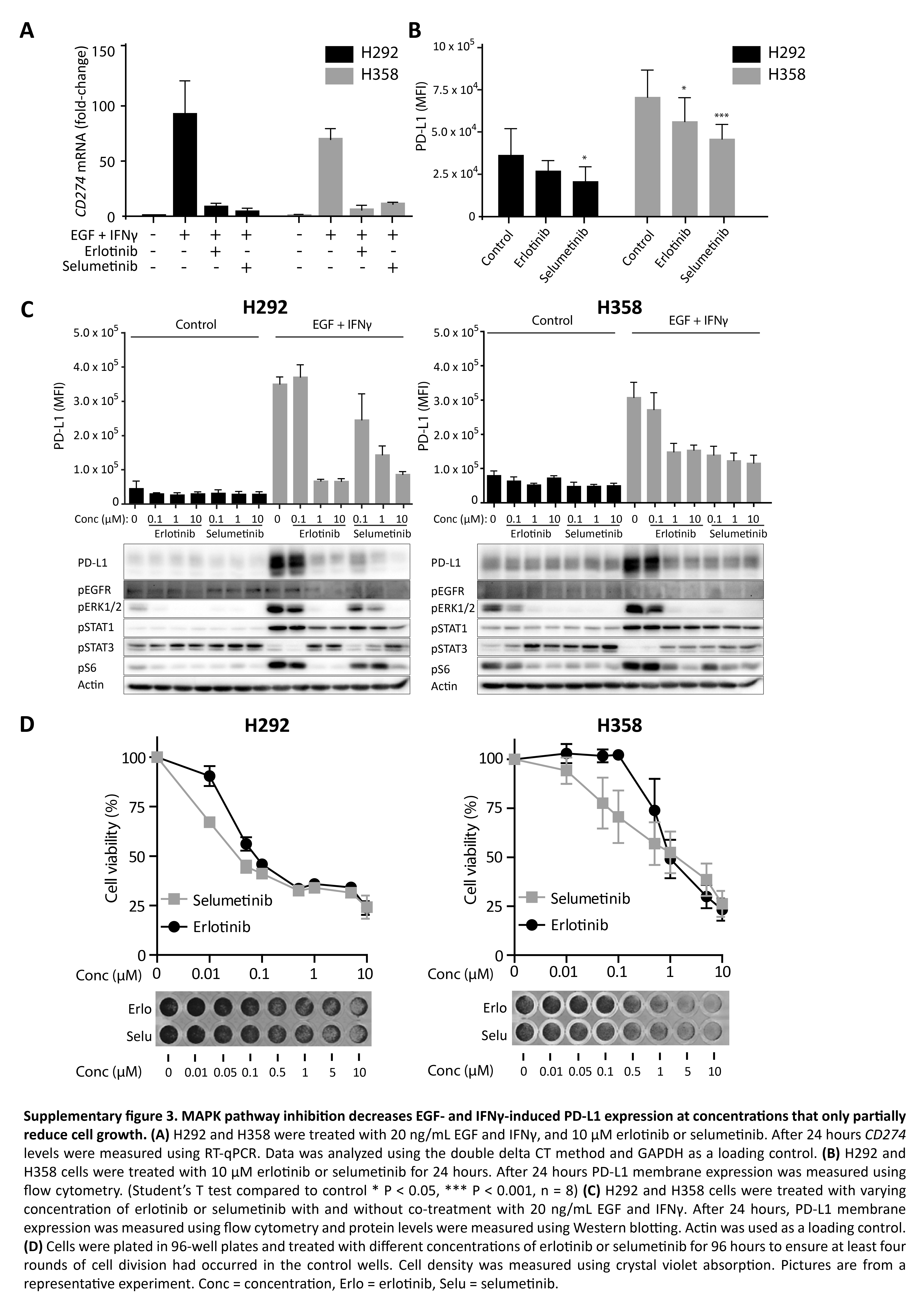

Supplement: Supplementary file 3 — Figure S3. MAPK pathway inhibition decreases EGF‐ and IFNγ‐induced PD‐L1 expression at concentrations that only partially reduce cell growth [file PATH-249-52-s003.tif]

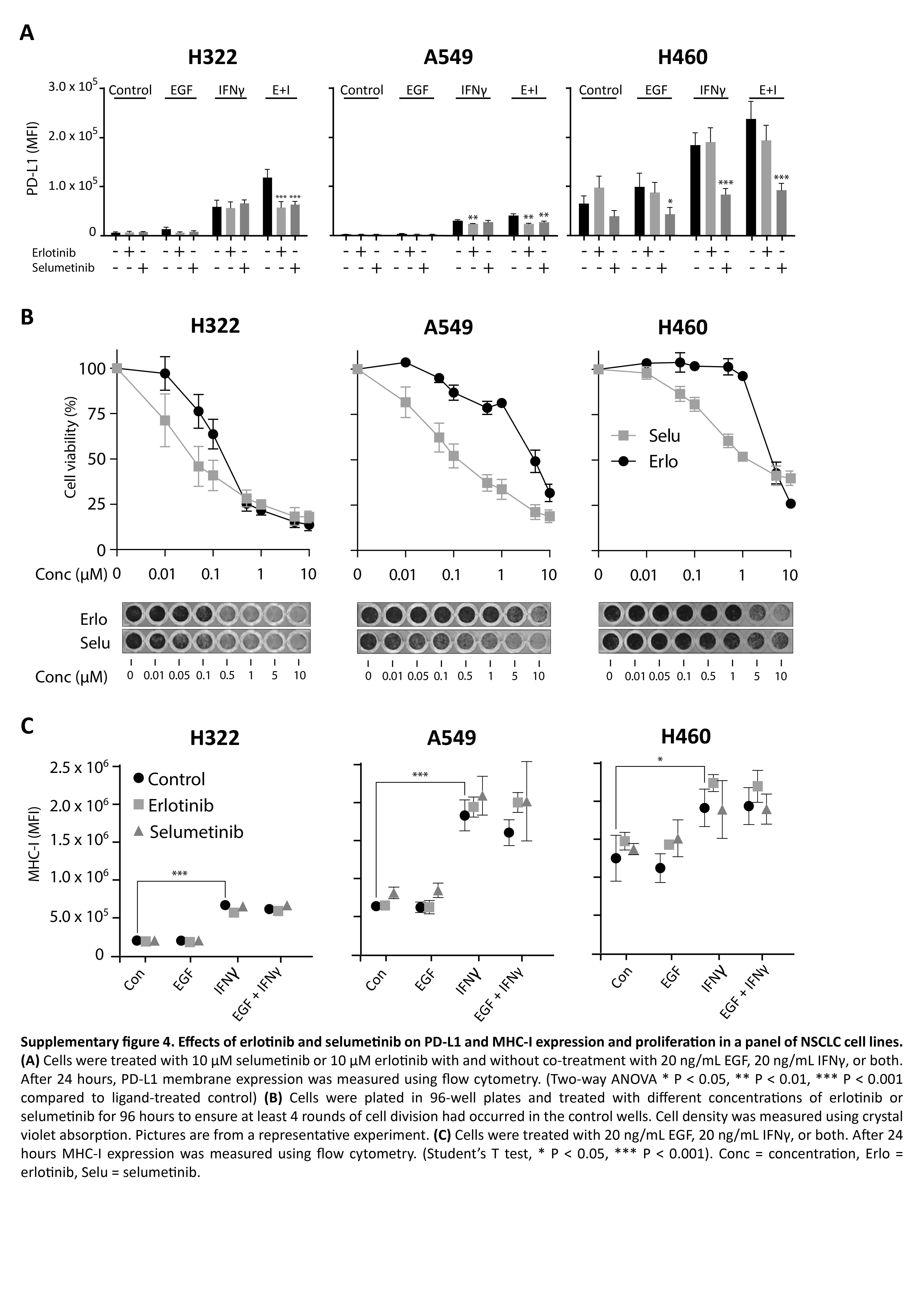

Supplement: Supplementary file 4 — Figure S4. Effects of erlotinib and selumetinib on PD‐L1 and MHC‐I expression and proliferation in a panel of NSCLC cell lines [file PATH-249-52-s004.tif]

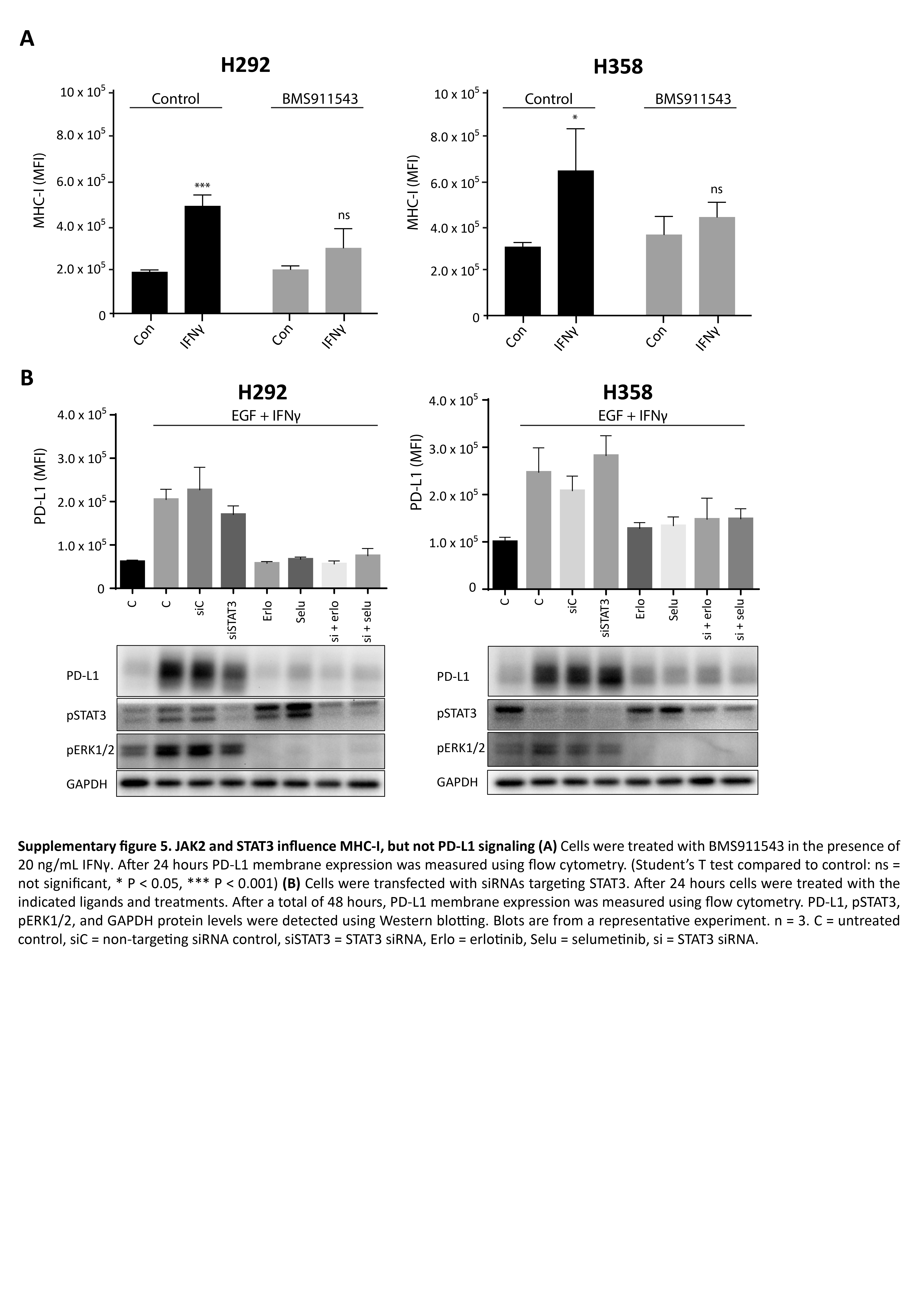

Supplement: Supplementary file 5 — Figure S5. JAK2 and STAT3 influence MHC‐I, but not PD‐L1 signaling [file PATH-249-52-s005.tif]

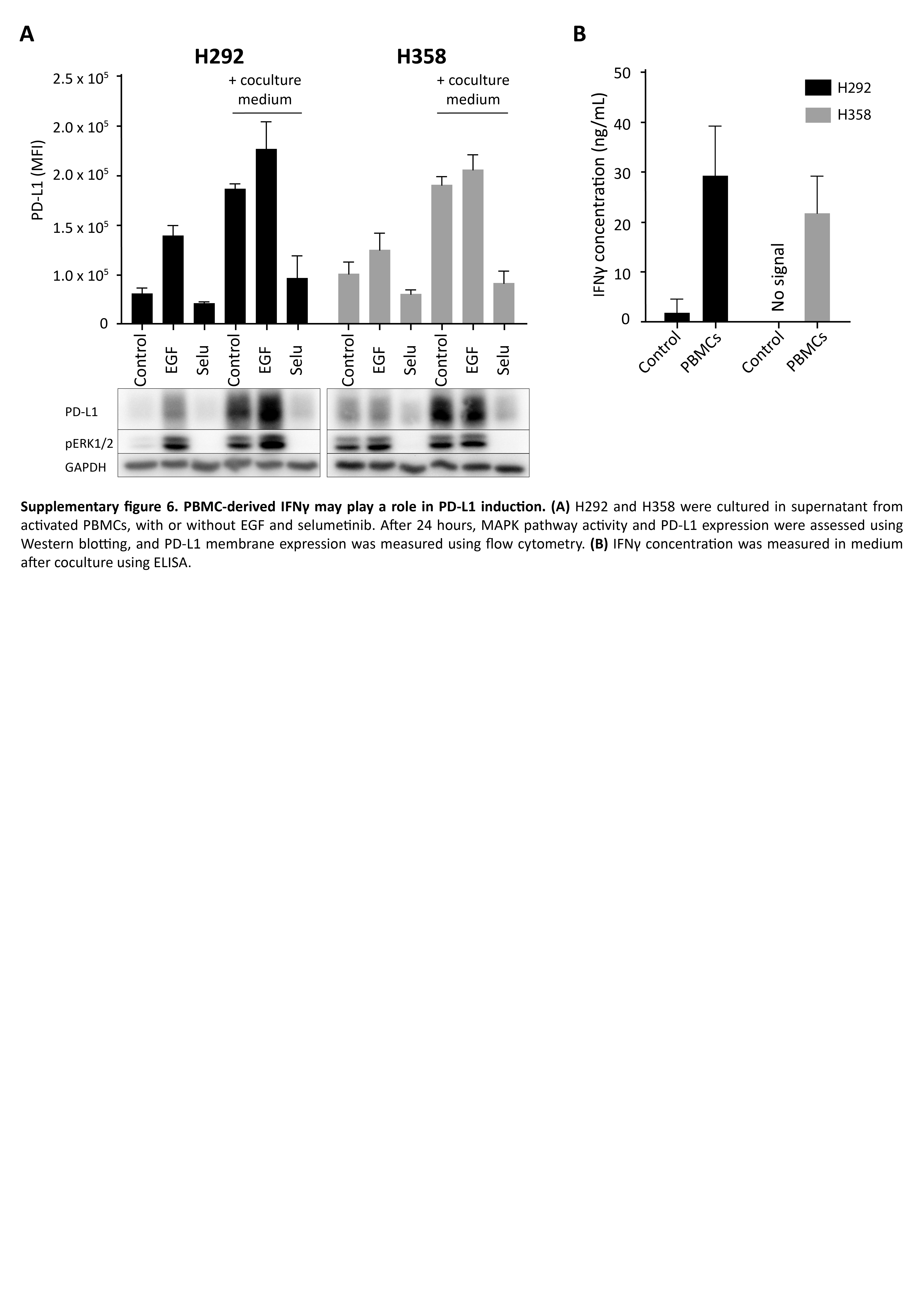

Supplement: Supplementary file 6 — Figure S6. PBMC‐derived IFNγ may play a role in PD‐L1 induction [file PATH-249-52-s006.tif]
