# Supplementary material for: MAPK pathway activity plays a key role in PD‐L1 expression of lung adenocarcinoma cells
Source: J Pathol. 2019 May 21;249(1):52–64. doi: 10.1002/path.5280 (PMC6767771; doi:10.1002/path.5280)
Supplement: Supplementary file 7 — Table S1. Antibodies used in the study [file PATH-249-52-s007.docx]

**MAPK pathway activity plays a key role in PD-L1 expression of lung adenocarcinoma cells**

**Stutvoet TS *et al*. J Pathol DOI: 10.1002/path.5280**

**Table S1.** Antibodies used in the study

| **Antibody** | **Dilutions** | **Catalog number/clone** | **Vendor** |
| --- | --- | --- | --- |
| *Flow cytometry* | | | |
| PD-L1 | 1:50 | 29E.2A3 | BioLegend (San Diego, CA, USA) |
| MHC-I | 1:50 | W6/32 | BioLegend |
| Anti-mouse-PE | 1:50 | 1030-09S | Southernbiotech (Birmingham, AL, USA) |
| *Western blot* | | | |
| EGFR | 1:250 | 2232 | Cell Signaling Technology (Danvers, MA, USA) |
| pEGFR | 1:500 | 3777 | Cell Signaling Technology |
| PD-L1 | 1:1000 | 13686 | Cell Signaling Technology |
| ERK | 1:1000 | 9102 | Cell Signaling Technology |
| pERK1/2 | 1:1000 | 9106 | Cell Signaling Technology |
| STAT1 | 1:1000 | 9172 | Cell Signaling Technology |
| pSTAT1_Ser727_ | 1:1000 | 8826 | Cell Signaling Technology |
| STAT3 | 1:1000 | 12640 | Cell Signaling Technology |
| pSTAT3_Tyr705_ | 1:1000 | 9145 | Cell Signaling Technology |
| AKT | 1:1000 | 9275 | Cell Signaling Technology |
| pAKT_Thr308_ | 1:1000 | 9271 | Cell Signaling Technology |
| S6 | 1:1000 | 2217 | Cell Signaling Technology |
| pS6 | 1:1000 | 2211 | Cell Signaling Technology |
| CMTM6 | 0.4 µg/ml | HPA026980 | Atlas Antibodies (Bromma, Sweden) |
| GAPDH | 1:1000 | 128915 | Abcam (Cambridge, UK) |
| β-actin | 1:10000 | 69100 | MP Biochemicals (Santa Ana, CA, USA) |
| HRP-anti-mouse | 1:1500 | P0260 | Dako (Glostrup, Denmark) |
| HRP-anti-rabbit | 1:1500 | P0448 | Dako |
