# Supplementary material for: MAPK pathway activity plays a key role in PD‐L1 expression of lung adenocarcinoma cells
Source: J Pathol. 2019 May 21;249(1):52–64. doi: 10.1002/path.5280 (PMC6767771; doi:10.1002/path.5280)
Supplement: Supplementary file 8 — Table S2. (A) Protein band sizes that were considered as specific staining for the target protein. (B) Quantitation of densitometry of all presented Western blotting results [file PATH-249-52-s008.docx]

**MAPK pathway activity plays a key role in PD-L1 expression of lung adenocarcinoma cells**

**Stutvoet TS *et al*. J Pathol DOI: 10.1002/path.5280**

**Table S2**

1. Protein band sizes that were considered as specific staining for the target protein

|  | |
| --- | --- |
| PD-L1 | 40–50 kDa |
| pEGFR | 180 kDa |
| EGFR | 175 kDa |
| pSTAT1 | 90 kDa |
| STAT1 | 90 kDa |
| pSTAT3 | 90 kDa |
| STAT3 | 90 kDa |
| pERK1/2 | 42/44 kDa |
| ERK1/2 | 42/44 kDa |
| pAKTS473 | 60 kDa |
| pAKTT308 | 60 kDa |
| AKT | 60 kDa |
| pS6 | 23 kDa |
| S6 | 23 kDa |
| Actin | 42 kDa |
| GAPDH | 38 kDa |

1. Quantitation of densitometry of all presented western blotting results

Figure 2E

| H292 | Control | 1 h  EGF | 24 h  EGF | 48 h  E + I | 72 h  EGF | 1 h  IFN | 24 h  IFN | 48 h  IFN | 72 h  IFN | 1 h  E + I | 24 h  E + I | 48 h  E + I | 72 h  E + I |
| --- | --- | --- | --- | --- | --- | --- | --- | --- | --- | --- | --- | --- | --- |
| PD-L1 | 1.00 | 1.76 | 3.42 | 4.17 | 4.84 | 1.53 | 2.54 | 4.91 | 5.61 | 1.39 | 6.74 | 8.50 | 8.17 |
| pERK1/2 | 1.00 | 2.48 | 2.40 | 1.87 | 2.01 | 0.66 | 0.27 | 0.69 | 1.15 | 2.00 | 1.74 | 1.44 | 0.95 |
| ERK1/2 | 1.00 | 0.87 | 0.98 | 0.75 | 1.02 | 1.10 | 0.80 | 0.78 | 0.85 | 0.81 | 0.99 | 0.92 | 0.88 |
| pSTAT1 | 1.00 | 4.52 | 2.48 | 2.03 | 2.34 | 6.17 | 6.11 | 5.82 | 4.96 | 5.62 | 8.74 | 10.14 | 8.79 |
| STAT1 | 1.00 | 1.45 | 1.31 | 1.17 | 1.29 | 1.50 | 3.78 | 3.58 | 2.70 | 1.39 | 3.65 | 3.52 | 3.21 |
| pSTAT3 | 1.00 | 0.56 | 0.23 | 0.14 | 0.15 | 0.93 | 0.44 | 0.05 | 0.04 | 0.25 | 0.03 | 0.06 | 0.12 |
| STAT3 | 1.00 | 1.82 | 1.48 | 1.16 | 1.21 | 1.47 | 1.23 | 1.21 | 0.97 | 1.00 | 1.30 | 1.19 | 1.31 |
| pS6 | 1.00 | 11.98 | 9.07 | 5.52 | 4.14 | 1.77 | 1.67 | 5.19 | 6.03 | 5.15 | 6.44 | 5.12 | 5.18 |
| S6 | 1.00 | 1.63 | 1.50 | 0.92 | 1.04 | 0.90 | 0.70 | 0.81 | 0.76 | 1.02 | 1.45 | 1.28 | 1.18 |

| H358 | Control | 1 h  EGF | 24 h  EGF | 48 h  E + I | 72 h  EGF | 1 h  IFN | 24 h  IFN | 48 h  IFN | 72 h  IFN | 1 h  E + I | 24 h  E + I | 48 h  E + I | 72 h  E + I |
| --- | --- | --- | --- | --- | --- | --- | --- | --- | --- | --- | --- | --- | --- |
| PD-L1 | 1.00 | 0.90 | 1.07 | 1.32 | 1.50 | 1.12 | 3.38 | 4.33 | 3.85 | 1.00 | 3.29 | 4.18 | 4.21 |
| pERK1/2 | 1.00 | 6.37 | 5.02 | 6.55 | 2.71 | 2.83 | 5.94 | 6.75 | 4.86 | 5.47 | 3.95 | 6.74 | 3.42 |
| ERK1/2 | 1.00 | 0.95 | 0.95 | 1.01 | 0.88 | 0.98 | 0.94 | 1.03 | 0.83 | 0.98 | 0.80 | 0.82 | 0.92 |
| pSTAT1 | 1.00 | 1.33 | 0.88 | 0.81 | 0.79 | 3.21 | 2.90 | 4.19 | 3.82 | 3.83 | 3.54 | 3.67 | 3.24 |
| STAT1 | 1.00 | 1.12 | 1.15 | 1.18 | 0.90 | 1.09 | 1.95 | 2.27 | 2.02 | 0.89 | 1.99 | 2.44 | 1.57 |
| pSTAT3 | 1.00 | 0.55 | 0.53 | 0.55 | 0.95 | 0.80 | 0.97 | 0.91 | 1.02 | 0.69 | 0.31 | 0.45 | 0.38 |
| STAT3 | 1.00 | 0.97 | 1.05 | 1.07 | 1.18 | 1.33 | 1.23 | 1.35 | 1.21 | 1.02 | 1.12 | 0.97 | 0.86 |
| pS6 | 1.00 | 2.64 | 2.37 | 1.93 | 1.15 | 2.28 | 2.26 | 1.53 | 1.43 | 1.51 | 0.95 | 0.98 | 1.23 |
| S6 | 1.00 | 1.00 | 1.07 | 1.11 | 1.16 | 1.30 | 1.05 | 1.58 | 1.43 | 1.40 | 1.11 | 0.87 | 0.76 |

Figure 3B

| H292 | Control | Control | Control | Control | Cetuximab | Cetuximab | Cetuximab | Cetuximab | Erlotinib | Erlotinib | Erlotinib | Erlotinib |
| --- | --- | --- | --- | --- | --- | --- | --- | --- | --- | --- | --- | --- |
|  | **Control** | **EGF** | **IFN** | **EGF + IFN** | **Control** | **EGF** | **IFN** | **EGF + IFN** | **Control** | **EGF** | **IFN** | **EGF + IFN** |
| PD-L1 | 1.00 | 1.70 | 2.47 | 5.64 | 1.23 | 0.91 | 1.74 | 3.17 | 0.92 | 0.66 | 1.45 | 1.93 |
| pEGFR | 1.00 | 0.26 | 0.47 | 0.15 | 1.58 | 2.13 | 2.53 | 1.82 | 0.04 | 0.03 | 0.06 | 0.06 |
| EGFR | 1.00 | 0.34 | 0.78 | 0.28 | 0.40 | 0.43 | 0.47 | 0.29 | 0.19 | 0.16 | 0.24 | 0.20 |
| pERK1/2 | 1.00 | 4.89 | 1.54 | 3.76 | 0.23 | 1.49 | 0.26 | 1.32 | 0.22 | 0.12 | 0.10 | 0.09 |
| ERK1/2 | 1.00 | 0.60 | 0.71 | 0.65 | 0.78 | 0.74 | 0.77 | 0.76 | 0.77 | 0.74 | 0.57 | 0.63 |
| pSTAT1 | 1.00 | 2.36 | 6.39 | 5.80 | 0.85 | 1.19 | 3.97 | 4.94 | 0.62 | 0.53 | 4.24 | 5.55 |
| STAT1 | 1.00 | 1.04 | 2.68 | 2.14 | 0.80 | 0.85 | 2.59 | 2.48 | 0.88 | 0.76 | 2.03 | 2.67 |
| pSTAT3 | 1.00 | 0.17 | 1.09 | 0.33 | 1.53 | 0.40 | 1.68 | 0.45 | 1.16 | 1.37 | 1.24 | 1.26 |
| STAT3 | 1.00 | 0.78 | 0.88 | 0.77 | 0.69 | 0.69 | 0.72 | 1.03 | 0.68 | 0.80 | 0.68 | 0.84 |
| pS6 | 1.00 | 3.12 | 2.37 | 12.15 | 0.89 | 1.85 | 1.07 | 10.50 | 0.67 | 0.29 | 0.34 | 0.55 |
| S6 | 1.00 | 0.79 | 0.79 | 0.71 | 0.78 | 0.75 | 0.70 | 0.78 | 0.56 | 0.75 | 0.57 | 0.56 |

| H358 | Control | Control | Control | Control | Cetuximab | Cetuximab | Cetuximab | Cetuximab | Erlotinib | Erlotinib | Erlotinib | Erlotinib |
| --- | --- | --- | --- | --- | --- | --- | --- | --- | --- | --- | --- | --- |
|  | **Control** | **EGF** | **IFN** | **EGF + IFN** | **Control** | **EGF** | **IFN** | **EGF + IFN** | **Control** | **EGF** | **IFN** | **EGF + IFN** |
| PD-L1 | 1.00 | 1.11 | 2.63 | 2.82 | 0.64 | 0.80 | 1.23 | 3.19 | 1.19 | 1.01 | 1.62 | 1.41 |
| pEGFR | 1.00 | 0.69 | 0.34 | 0.40 | 2.78 | 1.56 | 1.42 | 0.55 | 0.13 | 0.04 | 0.08 | 0.09 |
| EGFR | 1.00 | 0.87 | 0.91 | 0.71 | 0.97 | 1.07 | 0.98 | 1.06 | 1.26 | 1.01 | 1.14 | 0.87 |
| pERK1/2 | 1.00 | 1.23 | 0.34 | 0.72 | 0.51 | 1.25 | 0.22 | 0.75 | 0.14 | 0.09 | 0.11 | 0.15 |
| ERK1/2 | 1.00 | 0.89 | 0.88 | 1.04 | 1.13 | 1.13 | 0.97 | 0.95 | 1.22 | 1.07 | 1.08 | 0.91 |
| pSTAT1 | 1.00 | 1.29 | 3.92 | 4.34 | 1.15 | 1.38 | 3.09 | 3.94 | 1.11 | 1.17 | 4.76 | 3.99 |
| STAT1 | 1.00 | 1.08 | 2.66 | 2.57 | 0.78 | 0.75 | 1.72 | 1.58 | 0.65 | 0.61 | 1.88 | 2.01 |
| pSTAT3 | 1.00 | 0.57 | 0.15 | 0.14 | 0.96 | 0.39 | 0.61 | 0.25 | 2.35 | 2.59 | 2.59 | 1.56 |
| STAT3 | 1.00 | 0.95 | 1.03 | 1.08 | 0.99 | 1.01 | 0.98 | 0.99 | 1.12 | 1.16 | 1.33 | 1.23 |
| pS6 | 1.00 | 0.92 | 1.09 | 1.22 | 0.62 | 1.09 | 0.41 | 0.95 | 0.14 | 0.09 | 0.11 | 0.12 |
| S6 | 1.00 | 0.91 | 0.93 | 0.89 | 0.79 | 0.78 | 0.70 | 0.84 | 1.01 | 1.16 | 1.10 | 0.96 |

Figure 4B

| H292 | Control | Control | Control | Control | EGF | EGF | EGF | EGF | IFN | IFN | IFN | IFN | E + I | E + I | E + I | E + I |
| --- | --- | --- | --- | --- | --- | --- | --- | --- | --- | --- | --- | --- | --- | --- | --- | --- |
|  | **Control** | **XL147** | **Everolimus** | **Selu** | **Control** | **XL147** | **Everolimus** | **Selu** | **Control** | **XL147** | **Everolimus** | **Selu** | **Control** | **XL147** | **Everolimus** | **Selu** |
| PD-L1 | 1.00 | 1.11 | 1.38 | 0.48 | 3.52 | 1.56 | 1.89 | 0.64 | 1.82 | 2.90 | 4.05 | 3.38 | 10.01 | 7.55 | 8.12 | 3.17 |
| pERK1/2 | 1.00 | 1.00 | 1.18 | 0.10 | 2.96 | 1.71 | 1.73 | 0.11 | 0.48 | 0.50 | 0.70 | 0.07 | 1.82 | 1.56 | 1.89 | 0.14 |
| ERK1/2 | 1.00 | 1.14 | 1.01 | 1.26 | 1.48 | 0.91 | 0.86 | 1.08 | 1.10 | 1.12 | 1.04 | 1.10 | 0.75 | 0.71 | 0.66 | 0.70 |
| pSTAT1 | 1.00 | 1.14 | 1.10 | 1.40 | 2.06 | 1.10 | 1.02 | 0.57 | 3.33 | 3.95 | 3.98 | 4.13 | 5.50 | 5.57 | 4.89 | 2.56 |
| STAT1 | 1.00 | 0.94 | 0.76 | 0.91 | 0.94 | 0.74 | 0.53 | 0.64 | 2.53 | 2.51 | 2.48 | 2.84 | 2.06 | 2.69 | 1.80 | 2.33 |
| pSTAT3 | 1.00 | 1.61 | 1.27 | 1.84 | 0.34 | 0.37 | 0.30 | 1.28 | 1.04 | 1.47 | 1.51 | 1.87 | 0.19 | 0.29 | 0.38 | 1.17 |
| STAT3 | 1.00 | 1.10 | 0.96 | 1.23 | 0.96 | 0.56 | 0.42 | 0.50 | 0.69 | 0.63 | 0.71 | 0.76 | 0.82 | 0.62 | 0.63 | 0.68 |
| pAKTS473 | 1.00 | 1.01 | 1.48 | 1.12 | 3.97 | 0.72 | 1.52 | 3.84 | 2.67 | 0.63 | 2.02 | 2.48 | 6.11 | 1.55 | 1.90 | 4.65 |
| pAKTT308 | 1.00 | 0.45 | 1.22 | 0.97 | 1.42 | 0.22 | 0.56 | 1.33 | 0.79 | 0.14 | 0.88 | 0.57 | 2.22 | 0.34 | 1.21 | 1.77 |
| AKT | 1.00 | 1.41 | 1.03 | 1.48 | 1.07 | 0.82 | 0.61 | 0.70 | 0.77 | 0.73 | 0.75 | 0.80 | 0.78 | 0.80 | 0.66 | 0.58 |
| pS6 | 1.00 | 0.27 | 0.31 | 0.50 | 1.44 | 0.75 | 0.38 | 0.69 | 0.70 | 0.14 | 0.16 | 0.37 | 1.34 | 0.82 | 0.34 | 0.74 |
| S6 | 1.00 | 0.84 | 0.63 | 1.00 | 0.87 | 0.49 | 0.40 | 0.47 | 0.45 | 0.53 | 0.53 | 0.52 | 0.53 | 0.46 | 0.39 | 0.51 |

| H358 | Control | Control | Control | Control | EGF | EGF | EGF | EGF | IFN | IFN | IFN | IFN | E + I | E + I | E + I | E + I |
| --- | --- | --- | --- | --- | --- | --- | --- | --- | --- | --- | --- | --- | --- | --- | --- | --- |
|  | **Control** | **XL147** | **Everolimus** | **Selu** | **Control** | **XL147** | **Everolimus** | **Selu** | **Control** | **XL147** | **Everolimus** | **Selu** | **Control** | **XL147** | **Everolimus** | **Selu** |
| PD-L1 | 1.00 | 1.21 | 1.45 | 0.49 | 2.66 | 1.78 | 1.67 | 1.52 | 5.12 | 3.98 | 5.85 | 2.91 | 6.28 | 6.29 | 6.19 | 2.28 |
| pERK1/2 | 1.00 | 1.02 | 2.20 | 0.05 | 2.40 | 1.95 | 2.12 | 0.12 | 1.19 | 1.13 | 1.56 | 0.05 | 1.13 | 1.13 | 1.84 | 0.18 |
| ERK1/2 | 1.00 | 0.94 | 0.93 | 0.95 | 1.09 | 1.02 | 0.84 | 0.79 | 0.54 | 0.69 | 0.98 | 0.91 | 0.78 | 0.95 | 0.84 | 0.93 |
| pSTAT1 | 1.00 | 0.92 | 0.97 | 0.65 | 0.92 | 0.73 | 0.76 | 0.75 | 1.13 | 1.16 | 1.15 | 0.85 | 1.17 | 1.37 | 1.35 | 0.95 |
| STAT1 | 1.00 | 1.28 | 1.83 | 1.91 | 2.12 | 2.24 | 1.99 | 2.97 | 3.36 | 3.56 | 3.39 | 3.62 | 3.52 | 4.29 | 3.57 | 3.46 |
| pSTAT3 | 1.00 | 1.87 | 0.34 | 8.44 | 0.78 | 2.02 | 0.62 | 13.21 | 0.88 | 1.49 | 0.60 | 6.99 | 0.46 | 1.24 | 0.08 | 5.43 |
| STAT3 | 1.00 | 1.12 | 1.33 | 1.10 | 1.56 | 1.22 | 1.08 | 1.42 | 1.58 | 1.56 | 1.64 | 1.42 | 1.77 | 2.24 | 1.96 | 1.54 |
| pAKTS473 | 1.00 | 0.06 | 0.59 | 0.59 | 2.19 | 0.29 | 0.50 | 0.76 | 1.57 | 0.03 | 0.47 | 0.26 | 1.56 | 0.15 | 0.72 | 0.61 |
| pAKTT308 | 1.00 | 0.26 | 1.05 | 0.46 | 0.78 | 0.08 | 0.46 | 0.19 | 0.67 | 0.03 | 0.35 | 0.14 | 0.31 | 0.05 | 0.24 | 0.12 |
| AKT | 1.00 | 1.02 | 0.89 | 0.93 | 0.95 | 0.96 | 0.73 | 1.02 | 0.81 | 0.96 | 0.85 | 0.75 | 0.57 | 0.78 | 0.83 | 0.66 |
| pS6 | 1.00 | 0.58 | 0.38 | 0.29 | 1.13 | 0.70 | 0.30 | 0.51 | 1.02 | 0.62 | 0.28 | 0.43 | 0.77 | 0.72 | 0.43 | 0.58 |
| S6 | 1.00 | 1.16 | 1.09 | 0.94 | 1.63 | 1.20 | 0.96 | 1.17 | 1.15 | 1.06 | 1.02 | 1.27 | 1.19 | 1.27 | 0.73 | 0.82 |

Figure 5A

| H292 | Control | Control | Control | Control | BMS911543 | BMS911543 | BMS911543 | BMS911543 |
| --- | --- | --- | --- | --- | --- | --- | --- | --- |
|  | **Control** | **EGF** | **IFN** | **EGF + IFN** | **Control** | **EGF** | **IFN** | **EGF + IFN** |
| PD-L1 | 1.00 | 8.42 | 6.39 | 34.91 | 2.81 | 6.96 | 1.99 | 5.87 |
| pSTAT1 | 1.00 | 1.68 | 7.66 | 6.81 | 0.67 | 1.43 | 2.89 | 3.35 |

| H358 | Control | Control | Control | Control | BMS911543 | BMS911543 | BMS911543 | BMS911543 |
| --- | --- | --- | --- | --- | --- | --- | --- | --- |
|  | **Control** | **EGF** | **IFN** | **EGF + IFN** | **Control** | **EGF** | **IFN** | **EGF + IFN** |
| PD-L1 | 1.00 | 0.76 | 1.86 | 2.54 | 0.48 | 0.60 | 0.60 | 0.49 |
| pSTAT1 | 1.00 | 1.23 | 2.08 | 2.40 | 0.85 | 0.79 | 1.59 | 1.41 |

Figure S2B

| H292 |  | EGF | EGF | EGF | IFN | IFN | IFN | EGF + IFN | EGF + IFN | EGF + IFN |
| --- | --- | --- | --- | --- | --- | --- | --- | --- | --- | --- |
|  | **Control** | **5 min** | **15 min** | **60 min** | **5 min** | **15 min** | **60 min** | **5 min** | **15 min** | **60 min** |
| PD-L1 | 1.00 | 1.10 | 1.39 | 1.62 | 1.23 | 0.94 | 0.84 | 0.84 | 0.93 | 1.01 |
| pEGFR | 1.00 | 10.49 | 9.45 | 9.80 | 1.00 | 0.94 | 2.59 | 9.93 | 16.89 | 7.63 |
| pERK1/2 | 1.00 | 19.64 | 16.22 | 14.40 | 0.80 | 0.44 | 0.74 | 19.30 | 18.51 | 18.09 |
| pSTAT1 | 1.00 | 1.81 | 3.04 | 4.88 | 1.17 | 1.71 | 5.87 | 1.33 | 3.19 | 7.41 |
| pSTAT3 | 1.00 | 2.26 | 2.12 | 1.25 | 2.05 | 2.34 | 2.86 | 2.50 | 3.80 | 2.34 |

| H358 |  | EGF | EGF | EGF | IFN | IFN | IFN | EGF + IFN | EGF + IFN | EGF + IFN |
| --- | --- | --- | --- | --- | --- | --- | --- | --- | --- | --- |
|  | **Control** | **5 min** | **15 min** | **60 min** | **5 min** | **15 min** | **60 min** | **5 min** | **15 min** | **60 min** |
| PD-L1 | 1.00 | 1.64 | 1.85 | 2.15 | 2.00 | 1.51 | 1.57 | 1.82 | 1.93 | 1.69 |
| pEGFR | 1.00 | 3.95 | 3.43 | 2.53 | 0.73 | 0.43 | 0.63 | 2.95 | 4.45 | 2.00 |
| pERK1/2 | 1.00 | 8.34 | 6.94 | 3.32 | 2.24 | 1.05 | 1.05 | 7.31 | 6.46 | 1.76 |
| pSTAT1 | 1.00 | 1.45 | 1.67 | 2.08 | 1.35 | 1.09 | 2.13 | 1.22 | 1.47 | 3.00 |
| pSTAT3 | 1.00 | 1.41 | 1.19 | 0.95 | 1.31 | 1.18 | 1.08 | 1.51 | 1.59 | 0.58 |

Figure S3C

| H292 | Control | Control | Control | Control | Control | Control | Control | EGF + IFN | EGF + IFN | EGF + IFN | EGF + IFN | EGF + IFN | EGF + IFN | EGF + IFN |
| --- | --- | --- | --- | --- | --- | --- | --- | --- | --- | --- | --- | --- | --- | --- |
|  | **Control** | **Erlo** | **Erlo** | **Erlo** | **Selu** | **Selu** | **Selu** | **Control** | **Erlo** | **Erlo** | **Erlo** | **Selu** | **Selu** | **Selu** |
|  | **Control** | **0.1** | **1** | **10** | **0.1** | **1** | **10** | **Control** | **0.1** | **1** | **10** | **0.1** | **1** | **10** |
| PD-L1 | 1.00 | 0.90 | 0.96 | 1.05 | 0.72 | 0.85 | 1.08 | 11.10 | 9.53 | 2.61 | 2.52 | 3.65 | 1.69 | 0.69 |
| pEGFR | 1.00 | 0.84 | 0.85 | 0.31 | 0.79 | 0.70 | 1.46 | 1.58 | 1.73 | 0.91 | 0.55 | 0.63 | 0.74 | 0.70 |
| pERK1/2 | 1.00 | 0.11 | 0.01 | 0.01 | 0.08 | 0.07 | 0.05 | 5.14 | 3.79 | 0.30 | 0.06 | 2.82 | 1.62 | 0.07 |
| pSTAT1 | 1.00 | 1.11 | 0.86 | 1.23 | 1.14 | 1.31 | 2.25 | 27.30 | 22.93 | 13.54 | 14.96 | 18.87 | 16.78 | 12.64 |
| pSTAT3 | 1.00 | 0.82 | 1.05 | 1.01 | 1.15 | 1.42 | 1.71 | 0.47 | 0.17 | 1.43 | 1.75 | 0.35 | 0.79 | 2.28 |
| pS6 | 1.00 | 0.44 | 0.28 | 0.29 | 0.34 | 0.54 | 0.72 | 5.75 | 4.34 | 0.57 | 0.65 | 3.25 | 4.21 | 2.07 |

| H358 | Control | Control | Control | Control | Control | Control | Control | EGF + IFN | EGF + IFN | EGF + IFN | EGF + IFN | EGF + IFN | EGF + IFN | EGF + IFN |
| --- | --- | --- | --- | --- | --- | --- | --- | --- | --- | --- | --- | --- | --- | --- |
|  | **Control** | **Erlo** | **Erlo** | **Erlo** | **Selu** | **Selu** | **Selu** | **Control** | **Erlo** | **Erlo** | **Erlo** | **Selu** | **Selu** | **Selu** |
|  | **Control** | **0.1** | **1** | **10** | **0.1** | **1** | **10** | **Control** | **0.1** | **1** | **10** | **0.1** | **1** | **10** |
| PD-L1 | 1.00 | 0.87 | 0.85 | 0.90 | 0.77 | 0.94 | 0.99 | 2.88 | 3.21 | 1.41 | 1.18 | 1.23 | 1.25 | 1.44 |
| pEGFR | 1.00 | 2.12 | 2.29 | 1.62 | 1.74 | 0.93 | 1.64 | 1.91 | 2.06 | 1.50 | 1.27 | 2.51 | 1.08 | 0.89 |
| pERK1/2 | 1.00 | 0.49 | 0.14 | 0.05 | 0.05 | 0.03 | 0.08 | 1.83 | 1.54 | 0.24 | 0.11 | 0.24 | 0.17 | 0.25 |
| pSTAT1 | 1.00 | 1.05 | 0.93 | 0.73 | 0.78 | 0.86 | 1.00 | 3.57 | 4.23 | 3.21 | 2.93 | 2.65 | 2.42 | 2.27 |
| pSTAT3 | 1.00 | 2.07 | 4.99 | 4.13 | 4.00 | 4.64 | 5.31 | 0.36 | 0.59 | 2.39 | 2.99 | 2.67 | 3.61 | 4.16 |
| pS6 | 1.00 | 0.73 | 0.40 | 0.36 | 0.42 | 0.40 | 0.47 | 1.80 | 2.15 | 0.97 | 0.53 | 1.09 | 0.69 | 0.60 |

Figure S5B

| H292 | Control | EGF + IFN | EGF + IFN | EGF + IFN | EGF + IFN | EGF + IFN | EGF + IFN | EGF + IFN |
| --- | --- | --- | --- | --- | --- | --- | --- | --- |
|  | **Control** | **Control** | **siRNA control** | **STAT3 siRNA** | **Erlo** | **Selu** | **STAT3 siRNA**  **+ Erlo** | **STAT3 siRNA**  **+ Selu** |
| PD-L1 | 1.00 | 5.46 | 5.35 | 4.12 | 1.20 | 1.53 | 1.51 | 1.19 |
| pSTAT3 | 1.00 | 1.59 | 1.55 | 1.14 | 1.93 | 2.51 | 1.27 | 0.89 |
| pERK1/2 | 1.00 | 2.24 | 2.15 | 1.33 | 0.03 | 0.18 | 0.08 | 0.11 |

| H358 | Control | EGF + IFN | EGF + IFN | EGF + IFN | EGF + IFN | EGF + IFN | EGF + IFN | EGF + IFN |
| --- | --- | --- | --- | --- | --- | --- | --- | --- |
|  | **Control** | **Control** | **siRNA control** | **STAT3 siRNA** | **Erlo** | **Selu** | **STAT3siRNA**  **+ Erlo** | **STAT3 siRNA**  **+ Selu** |
| PD-L1 | 1 | 3.81 | 4.11 | 4.21 | 2.02 | 1.62 | 1.86 | 1.71 |
| pSTAT3 | 1.00 | 0.19 | 0.20 | 0.06 | 0.95 | 1.18 | 0.56 | 0.70 |
| pERK1/2 | 1.00 | 2.28 | 2.11 | 2.19 | 0.34 | 0.16 | 0.05 | 0.32 |

Figure S6A

| H292 | Control | Control | Control | Cond. medium | Cond. medium | Cond. medium |
| --- | --- | --- | --- | --- | --- | --- |
|  | **Control** | **EGF** | **Selu** | **Control** | **EGF** | **Selumetinib** |
| PD-L1 | 1.00 | 4.51 | 1.11 | 8.99 | 12.64 | 1.94 |
| pERK1/2 | 1.00 | 7.55 | 0.30 | 7.36 | 11.75 | 0.45 |

| H358 | Control | Control | Control | Cond. medium | Cond. medium | Cond. medium |
| --- | --- | --- | --- | --- | --- | --- |
|  | **Control** | **EGF** | **Selu** | **Control** | **EGF** | **Selumetinib** |
| PD-L1 | 1.00 | 1.17 | 0.56 | 3.04 | 2.80 | 0.63 |
| pERK1/2 | 1.00 | 1.45 | 0.08 | 1.65 | 1.62 | 0.05 |
