# Supplementary material for: MAPK pathway activity plays a key role in PD‐L1 expression of lung adenocarcinoma cells
Source: J Pathol. 2019 May 21;249(1):52–64. doi: 10.1002/path.5280 (PMC6767771; doi:10.1002/path.5280)
Supplement: Supplementary file 9 — Table S3. Gene set enrichment results [file PATH-249-52-s009.docx]

**MAPK pathway activity plays a key role in PD-L1 expression of lung adenocarcinoma cells**

**Stutvoet TS *et al*. J Pathol DOI: 10.1002/path.5280**

**Table S3.** Gene set enrichment results

| Subtype | Gene set | ES | NES | Nom p val. | FDR q val. | FWER p val. |
| --- | --- | --- | --- | --- | --- | --- |
| LUAD_nontarget | MsigDB_HALLMARK_IFNG_RESPONSE | 0.74 | 2.07 | 0.00 | 0.00 | 0.00 |
| LUAD_nontarget | LOBODA_RAS_UP | 0.69 | 2.06 | 0.00 | 0.00 | 0.00 |
| LUAD_nontarget | MsigDB_C6_EGFR_UP.V1_UP* | 0.54 | 1.94 | 0.00 | 0.00 | 0.01 |
| LUAD_nontarget | MsigDB_HALLMARK_PI3K_AKT_MTOR_SIGNALING | 0.42 | 1.72 | 0.00 | 0.02 | 0.07 |
| LUAD_nontarget | DRY_MEK_UP | 0.70 | 1.68 | 0.02 | 0.03 | 0.09 |
| LUAD_nontarget | MsigDB_C6_MEK_UP.V1_DN* | 0.41 | 1.61 | 0.03 | 0.04 | 0.15 |
| LUAD_nontarget | MsigDB_C6_EGFR_UP.V1_DN* | 0.26 | 1.13 | 0.27 | 0.35 | 0.74 |
| LUAD_nontarget | MsigDB_C6_MEK_UP.V1_UP* | 0.28 | 1.04 | 0.39 | 0.42 | 0.83 |
| LUAD_nontarget | LOBODA_RAS_DOWN | -0.42 | -1.17 | 0.29 | 0.48 | 0.68 |
| LUAD_nontarget | CREIGHTON_PI3K_UP* | 0.25 | 0.96 | 0.52 | 0.49 | 0.90 |
| LUAD_nontarget | CREIGHTON_PI3K_DOWN* | -0.19 | -0.86 | 0.71 | 0.61 | 0.94 |
|  |  |  |  |  |  |  |
| LUAD_nontarget_KRASmt | MsigDB_HALLMARK_IFNG_RESPONSE | 0.71 | 2.05 | 0.00 | 0.00 | 0.00 |
| LUAD_nontarget_KRASmt | LOBODA_RAS_UP | 0.51 | 1.53 | 0.08 | 0.09 | 0.25 |
| LUAD_nontarget_KRASmt | MsigDB_HALLMARK_PI3K_AKT_MTOR_SIGNALING | 0.39 | 1.55 | 0.02 | 0.09 | 0.22 |
| LUAD_nontarget_KRASmt | MsigDB_C6_MEK_UP.V1_DN* | 0.41 | 1.55 | 0.03 | 0.12 | 0.22 |
| LUAD_nontarget_KRASmt | CREIGHTON_PI3K_UP* | 0.36 | 1.38 | 0.12 | 0.13 | 0.44 |
| LUAD_nontarget_KRASmt | MsigDB_C6_EGFR_UP.V1_UP* | 0.38 | 1.41 | 0.10 | 0.13 | 0.40 |
| LUAD_nontarget_KRASmt | MsigDB_C6_EGFR_UP.V1_DN* | 0.27 | 1.15 | 0.23 | 0.26 | 0.73 |
| LUAD_nontarget_KRASmt | DRY_MEK_UP | 0.42 | 1.18 | 0.25 | 0.27 | 0.70 |
| LUAD_nontarget_KRASmt | LOBODA_RAS_DOWN | -0.39 | -1.09 | 0.36 | 0.32 | 0.78 |
| LUAD_nontarget_KRASmt | CREIGHTON_PI3K_DOWN* | -0.25 | -1.15 | 0.20 | 0.38 | 0.69 |
| LUAD_nontarget_KRASmt | MsigDB_C6_MEK_UP.V1_UP* | -0.31 | -1.16 | 0.26 | 0.74 | 0.68 |
|  |  |  |  |  |  |  |
| LUAD_nontarget_KRASwt | MsigDB_HALLMARK_IFNG_RESPONSE | 0.72 | 2.12 | 0.00 | 0.00 | 0.00 |
| LUAD_nontarget_KRASwt | MsigDB_C6_EGFR_UP.V1_UP* | 0.60 | 2.11 | 0.00 | 0.00 | 0.00 |
| LUAD_nontarget_KRASwt | LOBODA_RAS_UP | 0.70 | 2.00 | 0.00 | 0.00 | 0.00 |
| LUAD_nontarget_KRASwt | DRY_MEK_UP | 0.78 | 1.88 | 0.00 | 0.01 | 0.02 |
| LUAD_nontarget_KRASwt | MsigDB_C6_MEK_UP.V1_UP* | 0.40 | 1.50 | 0.05 | 0.08 | 0.25 |
| LUAD_nontarget_KRASwt | MsigDB_HALLMARK_PI3K_AKT_MTOR_SIGNALING | 0.36 | 1.51 | 0.02 | 0.09 | 0.23 |
| LUAD_nontarget_KRASwt | MsigDB_C6_MEK_UP.V1_DN* | 0.35 | 1.43 | 0.07 | 0.10 | 0.32 |
| LUAD_nontarget_KRASwt | CREIGHTON_PI3K_DOWN* | 0.20 | 0.92 | 0.58 | 0.53 | 0.92 |
| LUAD_nontarget_KRASwt | MsigDB_C6_EGFR_UP.V1_DN* | 0.22 | 0.93 | 0.55 | 0.57 | 0.91 |
| LUAD_nontarget_KRASwt | CREIGHTON_PI3K_UP* | -0.20 | -0.80 | 0.68 | 0.71 | 0.97 |
| LUAD_nontarget_KRASwt | LOBODA_RAS_DOWN | -0.31 | -0.85 | 0.60 | 1.00 | 0.94 |
|  |  |  |  |  |  |  |
| LUAD_EGFRmt | MsigDB_HALLMARK_IFNG_RESPONSE | 0.65 | 1.87 | 0.01 | 0.01 | 0.01 |
| LUAD_EGFRmt | MsigDB_HALLMARK_PI3K_AKT_MTOR_SIGNALING | 0.38 | 1.58 | 0.01 | 0.07 | 0.18 |
| LUAD_EGFRmt | MsigDB_C6_EGFR_UP.V1_UP* | 0.41 | 1.55 | 0.04 | 0.07 | 0.22 |
| LUAD_EGFRmt | LOBODA_RAS_UP | 0.54 | 1.59 | 0.06 | 0.08 | 0.17 |
| LUAD_EGFRmt | CREIGHTON_PI3K_UP* | 0.32 | 1.27 | 0.18 | 0.23 | 0.61 |
| LUAD_EGFRmt | MsigDB_C6_MEK_UP.V1_DN* | 0.28 | 1.21 | 0.18 | 0.25 | 0.69 |
| LUAD_EGFRmt | DRY_MEK_UP | 0.37 | 0.96 | 0.51 | 0.50 | 0.93 |
| LUAD_EGFRmt | MsigDB_C6_MEK_UP.V1_UP* | -0.25 | -0.98 | 0.47 | 0.63 | 0.92 |
| LUAD_EGFRmt | CREIGHTON_PI3K_DOWN* | -0.18 | -0.78 | 0.76 | 0.73 | 0.99 |
| LUAD_EGFRmt | MsigDB_C6_EGFR_UP.V1_DN* | -0.23 | -0.99 | 0.48 | 0.93 | 0.92 |
| LUAD_EGFRmt | LOBODA_RAS_DOWN | -0.44 | -1.19 | 0.30 | 1.00 | 0.72 |
|  |  |  |  |  |  |  |
| LUSC_nontarget | MsigDB_HALLMARK_IFNG_RESPONSE | 0.69 | 1.93 | 0.01 | 0.02 | 0.01 |
| LUSC_nontarget | MsigDB_HALLMARK_PI3K_AKT_MTOR_SIGNALING | 0.34 | 1.39 | 0.05 | 0.35 | 0.40 |
| LUSC_nontarget | MsigDB_C6_MEK_UP.V1_DN* | 0.33 | 1.29 | 0.16 | 0.40 | 0.53 |
| LUSC_nontarget | MsigDB_C6_EGFR_UP.V1_UP* | 0.34 | 1.17 | 0.30 | 0.51 | 0.71 |
| LUSC_nontarget | MsigDB_C6_MEK_UP.V1_UP* | -0.22 | -0.77 | 0.75 | 0.76 | 0.99 |
| LUSC_nontarget | CREIGHTON_PI3K_UP* | 0.20 | 0.83 | 0.69 | 0.83 | 0.98 |
| LUSC_nontarget | LOBODA_RAS_DOWN | 0.33 | 0.95 | 0.51 | 0.83 | 0.94 |
| LUSC_nontarget | LOBODA_RAS_UP | 0.23 | 0.65 | 0.79 | 0.88 | 1.00 |
| LUSC_nontarget | DRY_MEK_UP | 0.36 | 0.85 | 0.63 | 0.91 | 0.97 |
| LUSC_nontarget | CREIGHTON_PI3K_DOWN* | 0.14 | 0.65 | 0.99 | 0.98 | 1.00 |
| LUSC_nontarget | MsigDB_C6_EGFR_UP.V1_DN* | -0.20 | -0.85 | 0.73 | 1.00 | 0.98 |

ES = Enrichment score

NES = Normalized enrichment score

Nom p val. = nominal *P* value

FDR q val. = False discovery rate q value

FWER p val. = Familywise error rate *P* value

LUAD_EGFRmt = EGFR mutant lung adenocarcinoma

LUAD_nontarget = lung adenocarcinoma without targetable driver mutations

LUAD_nontarget_KRASmt = lung adenocarcinoma without targetable driver mutations with KRAS mutations

LUAD_nontarget_KRASwt = lung adenocarcinoma without targetable driver mutations without KRAS mutations

LUSC_nontarget = squamous cell lung carcinoma without targetable driver mutations

*Signatures were derived from experiments in a single breast cancer cell line and may only partially

reflect activity of the target pathway in NSCLC
